# Tissue Doppler Imaging for anthracycline cardiotoxicity monitoring in pediatric patients with cancer

**DOI:** 10.1186/s40959-018-0032-3

**Published:** 2018-09-03

**Authors:** Francesco Venturelli, Riccardo Masetti, Marianna Fabi, Roberto Rondelli, Anna Martoni, Arcangelo Prete, Marco Bonvicini, Andrea Pession

**Affiliations:** 1 Department of Pediatrics “Lalla Seràgnoli”, S.Orsola-Malpighi Hospital, Hematology-Oncology Unit, University of Bologna, Bologna, Italy; 2 Pediatric Cardiology and Adult Congenital Unit, S.Orsola-Malpighi Hospital, University of Bologna, Bologna, Italy

**Keywords:** Anthracyclines, Cardiotoxicity, Echocardiography, Tissue Doppler imaging

## Abstract

**Background:**

Cardiotoxic effects of anthracycline therapy are a major cause of morbidity for childhood cancer survivors. The aim of this retrospective evaluation is to assess the efficacy of Tissue Doppler Imaging in the early detection of myocardial alterations in these patients.

**Methods:**

A population of 50 childhood cancer survivors (32 males and 18 females) who have been treated with anthracyclines was evaluated by standard and TDI echocardiographic examination of the basal and median region of the interventricular septum (IVSb, IVSm), of the left ventricular posterior wall (LVPWb, LVPWm), and of the mitral annulus; the results were compared with those obtained from a population of 50 healthy age-matched and sex-matched controls by using the Student test. The clinical and echocardiographic data of the two groups were compared also with the independent samples t-test. All data were expressed as mean ± standard deviation. A two-tailed *P*-value < 0.05 was considered statistically significant. Statistical analysis was performed using STATA 7.0.

**Results:**

The case-control analysis showed statistically significant differences (*p* < 0,05) between the patients and the controls values. The systolic performance of the patients was normal (LVEF (*p* = 0,0029) and LVFS (*p* = 0,0002)). Statistically significant differences between patients and controls were found for diastolic function measurements obtained with PW Doppler such as IVRT (*p* = 0,0000), DT (*p* = 0,0041), E (*p* = 0,0000), A (*p* = 0,0458), even if E/A ratio was not altered. TDI analysis also show significant differences between patients and controls in both LVPW and IVS (basal and middle segments); E/E’ ratio and E’/A’ ratio did not vary significantly. Linear Regression and multivariate analysis showed that Hematopoietic Stem Cell Transplantation had the highest impact on our measurements.

**Conclusions:**

The results showed a myocardial diastolic impairment with preserved ejection fraction. Since the median follow-up time of our cohort was 2 years, further evaluation is needed to better define the diastolic alterations. TDI analysis showed high sensitivity for the detection of mild myocardial dysfunction; the implementation of this novel method as standard practice in the follow-up of selected childhood cancer survivors might help to achieve a better management of long-term complications of cardiotoxic chemotherapy.

**Electronic supplementary material:**

The online version of this article (10.1186/s40959-018-0032-3) contains supplementary material, which is available to authorized users.

## Background

Anthracycline-induced cardiotoxicity represents the most important therapeutic limitation for both children and adults with neoplasia [1]. Childhood cancer survivors have an high risk of developing a progressive myocardial dysfunction, which has a major impact on their quality of life and survival [2]. Anthracycline antibiotics act through various molecular mechanisms, such as the inhibition of both α and β isozymes of the Topoisomerase II enzyme, the structural and functional alteration of the mithocondrion and the production of reactive species of oxygen (ROS). Their interference with intracellular signalling pathways eventually leads to cell death [3]. The myocardium is more susceptible than other tissues to the action of these antineoplastic agents because it expresses high levels of Topoisomerase IIβ and it is less resistant to oxidative stress. It is also well established that anthracyclines-related myocardial damage is influenced by specific patient-related factors such as cumulative dose of anthracyclines, female gender, age at diagnosis, mediastinal irradiation and tumor type [1, 4]. Their toxicity ultimately lead to a progressive chronic cardiomyopathy, which tipically involves the left chambers of the heart: while systolic function is usually preserved, a mild diastolic dysfunction can be detected. Long-term cardiac monitoring is necessary to reveal and quantify these myocardial alterations. Standard echocardiographic evaluation represents the best screening method because of its low cost and high diffusion; however its sensitivity toward mild cardiac dysfunction might be improved, especially in the early stages of the follow-up.

Pulsed-wave Tissue Doppler Imaging (TDI) is a non-invasive tecnique for the evaluation of myocardial tissue velocities; this study aims to evaluate the role of TDI in the early detection of the mild subclinical dysfunction induced by anthracyclines.

## Methods

A retrospective evaluation was performed on survivors of childhood cancer treated with anthracyclines at the Pediatric Hematology-Oncology Unit of the S.Orsola-Malpighi Clinic. All children were evaluated with standard echocardiography and Tissue Doppler Imaging (TDI) using iE 33 xMatrixs Echo system (Philips, Eindhoven, The Netherlands). The results were compared with those obtained from a population of healthy age-matched and sex-matched controls.

The patients cohort includes 50 children (32 males, 18 females), mean age of 11,7 (±4,7 SD) years, with mean body surface area (BSA) of 1,35 (±0,32 SD) m^2^. They were treated for leukemia (*n* = 31), lymphoma (*n* = 15), and other solid tumors (*n* = 4), with a median cumulative dose of 219,6 (range 83–780) mg/m^2^ of anthracyclines. The mean age at the start of chemotherapy was (7,7 ± 4,7 SD) years, with a median follow-up period of 24 months (4–127 months) after the anthracycline treatment was completed. Among these, 14 children (8 male, 6 females) underwent haematopoietic stem cell transplantation and 11 children received mediastinal irradiation (7 males, 4 females). The control population includes 50 subjects (30 males, 20 females) with a mean age of 11,5 (± 5,4 SD) years and mean body surface area of 1,31 (±0,39 SD).

The cumulative doses of anthracyclines were converted into Doxorubicin (DOX) equivalents, using conversion factors of 0,83 (Daunorubicin), 5,0 (Idarubicin) and 4,0 (Mitoxantrone) [5].

An expert pediatric cardiologist and a trained pediatrician performed the transthoracic echocardiographies. All measurements were performed on 3 consecutive cardiac cycles. The pediatric cardiologist reviewed all the images acquired. The analysis included standard left ventricle chamber measurements (two-dimensional M-mode), blood flow velocity through the mitral valve (pulsed-wave doppler) and tissue peak velocities for the interventricular septum and left ventricle parietal wall (pulsed-wave TDI).

The variables measured on 2D M-mode ultrasonography were: interventricular septum thickness in diastole (IVSd) and systole (IVSs), left ventricle end-diastolic diameter (LVEDD or LVIDd), left ventricle end-systolic diameter (LVESD or LVIDs), left ventricle posterior wall in diastole (LVPWd) and systole (LVPWs). Shortening fraction (SF) and ejection fraction (EF) were calculated from M-mode measurements of LV dimensions at the level of mitral valve leaflet using parasternal long axis view. The EF was considered normal when ≥55% and mildly abnormal between 54 and 45% [6]. LVFS was considered normal when ≥28%. LVEF and LVSF are good surrogate measure of systolic performance, while ventricular (LVIDd/LVIDs and LVPWd/LVPWs) and septal (IVSd and IVSs) dimensions were used to monitor the impact of the treatment on the ventricular structure [5]. Z-scores were calculated for M-mode parameters (IVSs, LVIDs, LVIDd, LVPWd). Despite susceptible of both inter- and intra-rater variability, these measures are easily reproducible, can be monitored over time and was found to be reliable in pediatric patients [7].

**Fig. 1:**
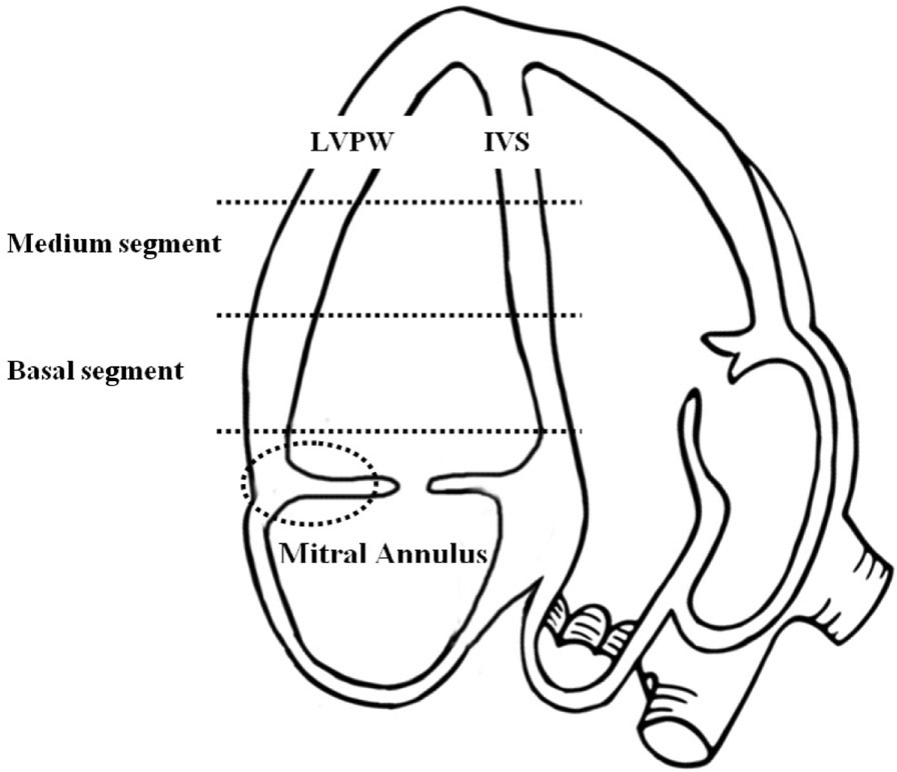
Pulsed-wave TDI sample volume placement regions

Pulsed-wave Doppler was used to evaluate the diastolic function: mitral inflow wave velocity in the early diastole (E) and in the late diastole (A), left ventricle isovolumic relaxation time (IVRT) and E deceleration time (DT) were measured. Sample volume was placed between the leaflets of the mitral valve. The Diastolic function was considered normal when E/A ratio ranged between 0.9 and 1.5, abnormal with delayed relaxation pattern if E/A ratio < 0.9 and with restrictive pattern if E/A ratio > 2. Pulsed-wave Tissue Doppler Imaging (TDI) was used to add information on both systolic and diastolic performance. The apical four chamber view was used to assess peak myocardial velocities of the LV during systole (S′), early diastole (E’) and late diastole (A’). E’/A’ index and E/E’ index were calculated. The sample volume was placed at the basal and median segments of the interventricular septum (IVSb, IVSm) and left ventricular posterior wall (LVPWb, LVPWm). We did not include the evaluation of LV apical segments because myocardial velocities decreases toward the apex of the heart, resulting in unreliable and less significant measurements in this segment [8]. The same measurements were recorded from the lateral segment of the mitral annulus to evaluate the mitral annular displacement (MAD), as suggested from the American Society of Echocardiography [9] (Fig. 1). We chose to evaluate these three segments to detect potential regional wall motion abnormalities [10].

The patient’s cohort was divided into sub-groups according to risk factors for anthracycline cardiomyopathy [1, 4]: A) *Total cumulative dose of anthracyclines* the patients’ cohort was divided into 3 subgroups according to the literature [11]: 1)low-risk: < 150 mg/m^2^; 2)medium-risk: 150–300 mg/m^2^; 3)high-risk: > 300 mg/m^2^. We compared the low-risk class with patients at medium- and high-risk, and the high-risk class with patients at low- and medium-risk. B) *Gender* C) *Medistinic Irradiation* D) *Haematopoietic stem cell transplantation (HSCT)*: the results obtained from the children who underwent HSCT at the Pediatric Hematology-Oncology Unit of the S.Orsola-Malpighi Clinic were compared with those who didn’t receive such procedure during their therapy. E) *Oncologic diagnosis*. F) *Follow-up duration at the time of the evaluation*: we compared the patients evaluated within 12 months after the end of therapy with those evaluated after 12 months after the end of therapy. The same comparison was carried out using also a 24 months cut-off.

Analysis of variance was used to assess differences in the mean value of the normally distributed variables between groups, while the Student Test was used for within-group post-hoc analysis. A *p* value < 0.05 was considered to be statistically significant.

The statistically significant measurements of the case-control analysis were analyzed by linear regression analysis; multivariate analysis was also performed to determine the influence of the risk factors on each parameters. The independent variables considered were 1) time from end of therapy to TDI evaluation, 2) time from diagnosis to TDI evaluation, 3) gender, 4) BSA (body surface area), 5) anthracycline equivalent dose, 6) Hematopoietic Stem Cell Transplantation procedure, 7) Tumor Type. A *p* value < 0.05 was considered to be statistically significant. The clinical and echocardiographic data between the two groups were compared by the independent-samples t-test. All data were expressed as mean ± standard deviation. A two-tailed *P*-value < 0.05 was considered statistically significant. Statistical analysis was performed using STATA 7.0.

## Results

### Case-control analysis

Table 1 shows the comparison of 50 survivors of childhood cancer and “healthy controls”. The IVS was significantly bigger in both diastole (*p* = 0,0000) and systole (*p* = 0,0186) of the treated children. LVPWd showed a significant thickness increment (*p* = 0,0000) as opposed to LVPWs. The patients cohort showed a significant decrement of both LVSF (*p* = 0,0002) and LVEF (*p* = 0,0029), but values remained in the normal range (LVEF ≥55%, LVSF ≥28%). Z-score for the M-mode values of the patients showed that both IVSd (2,06) and LVPWd (1,05) were higher than the European children population reference values, while LVIDd (− 0,57) was lower and LVIDs (0,08) was in line with the reference values. The PW Doppler analysis of the transmitral blood flow revealed that the E deceleration time of the patients was significantly reduced (*p* = 0,0041) as well as E (*p* = 0,0000) and A (*p* = 0,0458) peak velocities values. IVRT was significantly higher in patients (*p* = 0,0000) while E/A ratio was lower, but with a *p* value > 0,05. TDI analysis of the LVPW middle segment showed a significant reduction of both E’ (*p* = 0,0004) and A’ (*p* = 0,0111) among patients, while S (*p* = 0,0000) was increased. Both E’/A’ ratio increment (*p* = 0,6022) and E/E’ ratio decrement (*p* = 0,2322) were not significant. LVPW basal segment revealed a similar profile, with higher S (*p* = 0,0132), lower E’ (*p* = 0,0001) and lower A’ (*p* = 0,0006) values compared to controls. E’/A’ ratio and E/E’ ratio didn’t show statistically significant alterations. E’ showed a statistically significant reduction (*p* = 0,0269) in the IVS middle segment only, while the IVS basal segment analysis revealed a significant reduction of S (*p* = 0,0031), E’(*p* = 0,0045) and A’ (*p* = 0,0178). E’/A’ ratio and E/E’ ratio values alterations were not significant. Mitral annular displacement measures showed significant alterations of both E’ (*p* = 0,0002) and A’ (*p* = 0,0219). Both our standard PW Doppler and TDI measurements revealed significant diastolic alterations in the patients’ cohort, but they did not reflect any typical diastolic dysfunction pattern; in fact E/A, E/E’ and E’/A’ (the most important values to characterize diastolic anomalies)(8), didn’t vary significantly between patients and controls.

**Table 1:** T-test results between patient cohort and control cohort (*p* < 0,05)

	PATIENTS (*n* = 50)	CONTROLS (*n* = 50)	PATIENTS Z-score	*P* values
	mean values (± SD)	mean values (± SD)	mean values (± SD)	
IVSd (cm)	0.863 (±0.144)	0.746 (±0.113)	2.06 (±1.35)	0.0000
IVSs (cm)	1.153 (±0.248)	1.047 (±0.189)		0.0186
LVIDd (cm)	3.995 (±0.519)	4.104 (±0.586)	−0.57 (±1.32)	0.3290
LVIDs (cm)	2.694 (±0.435)	2.584 (±0.417)	0.08 (±1.27)	0.2011
LVPWd (cm)	0.813 (±0.165)	0.678 (±0.151)	1.05 (±0.95)	0.0000
LVPWs (cm)	1.106 (±0.240)	1.070 (±0.228)		0.4460
FS (%)	32.52 (±6.28)	36.95 (±5.15)		0.0002
FE (%)	61.79 (±0.10)	66.99 (±0.07)		0.0029
DECEL TIME (s)	0.173 (±0.068)	0.210 (±0.044)		0.0041
IVRT (s)	0.096 (±0.025)	0.061 (±0.009)		0.0000
E (m/s)	0.865 (±0.150)	1.035 (±0.172)		0.0000
A (m/s)	0.493 (±0.111)	0.535 (±0.094)		0.0458
E/A RATIO	1.83 (±0.42)	1.99 (±0.43)		0.0732
LVPWm				
S (m/s)	0.096 (±0.027)	0.069 (±0.022)		0.0000
E’ (m/s)	0.155 (±0.030)	0.174 (±0.022)		0.0004
A’ (m/s)	0.045 (±0.013)	0.052 (±0.013)		0.0111
E’/A’ RATIO	3.701 (±1.219)	3.583 (±1.028)		0.6022
E/E’ RATIO	5.691 (±1.414)	5.992 (±0.982)		0.2322
LVPWb				
S (m/s)	0.101 (±0.028)	0.089 (±0.016)		0.0132
E’ (m/s)	0.181 (±0.023)	0.207 (±0.039)		0.0001
A’ (m/s)	0.054 (±0.017)	0.066 (±0.017)		0.0006
E’/A’ RATIO	3.671 (±1.078)	3.336 (±0.969)		0.1047
E/E’ RATIO	4.791 (±0.984	5.107 (±1.054)		0.1326
IVSm				
S (m/s)	0.059 (±0.010)	0.058 (±0.009)		0.7279
E’ (m/s)	0.106 (±0.024)	0.115 (±0.018)		0.0269
A’ (m/s)	0.042 (±0.012)	0.045 (±0.009)		0.1438
E’/A’ RATIO	2.705 (±0.864)	2.678 (±0.614)		0.8569
E/E’ RATIO	8.501 (±2.525)	9.122 (±1.802)		0.1729
IVSb				
S (m/s)	0.074 (±0.014)	0.081 (±0.011)		0.0031
E’ (m/s)	0.128 (±0.023)	0.141 (±0.021)		0.0045
A’ (m/s)	0.049 (±0.013)	0.054 (±0.011)		0.0178
E’/A’ RATIO	2.830 (±0.938)	2.684 (±0.616)		0.3578
E/E’ RATIO	6.913 (±1.884)	7.501 (±1.703)		0.1116
MAD				
S (m/s)	0.077 (±0.021)	0.077 (±0.018)		0.9628
E’ (m/s)	0.145 (±0.031)	0.169 (±0.033)		0.0002
A’ (m/s)	0.050 (±0.020)	0.059 (±0.018)		0.0219
E’/A’ RATIO	3.405 (±1.732)	3.065 (±0.930)		0.2307
E/E’ RATIO	6.094 (±1.800)	6.392 (±2.292)		0.4873

*MAD* mitral annular displacement, *LVPWm* middle left ventricular posterior wall, *LVPWb* basal left ventricular posterior wall, *IVSm* middle interventricular septum, *IVSb* basal interventricular septum

### Subgroups T-test results

T-Test analysis between sub-groups of the patients’ cohort was performed to assess which parameter, if any, is the most sensitive to identify a cardiac alteration in a patient with a certain risk factor; an additional file shows the complete results (see Additional file 1). When taking the total cumulative dose of anthracyclines into consideration, the low-risk subjects did not show significant variations of TDI measurements compared to medium- and high-risk subjects (Additional file 1: Table S2). The values from the high-risk group did not vary significantly either, except for the E/E’ ratio variation (*p* = 0,0433) on the LVPW basal segment (Additional file 1: Table S3). The comparison between E/E’ ratios of the IVSm was not significant, but close to the cut-off (*p* = 0,0508). No statistically significant difference of TDI values was found comparing the gender groups (Additional file 1: Table S4), and comparing the mediastinal irradiation groups, with the exception of the atrial systole component of the Doppler transmitral pattern (A), which shows a significant increment in those who received mediastinal irradiation (Additional file 1: Table S5). Although the atrial systole component of the diastole can increase to compensate for poor left ventricle filling [12], its isolate alteration is not specific of any particular myocardial alteration. Patients who underwent HSCT during their treatment showed a significant decrease in E’ (*p* = 0,0303) of the basal segment of LVPW. Also the IVS analysis showed significant middle segment (E’, E’/A’ and E/E’) and basal segment (E’, A’ and E’/A’) alterations, while mitral annular displacement values were not significant (Additional file 1: Table S6). No statistical differences of TDI values were found between tumor type groups (Additional file 1: Table S7).

The patients evaluated after 12 months from the end of therapy were found to have a lower EF (*p* = 0,0486) than those evaluated before (Additional file 1: Table S8), while the patients evaluated after 24 months from the end of therapy had an higher IVRT (*p* = 0,0216) than those evaluated before (Additional file 1: Table S9).

### Linear regression and multivariate analysis

Linear regression analysis and Multivariate analysis was performed to asses which risk factor, if any, has the biggest impact on a particular measurement; an additional file shows the complete results (see Additional file 2). Hematopoietic Stem Cell Transplantation (HSCT) was found to be the most important risk factor. HSCT influenced the E’ wave of the LVPWb on both linear regression (*p* = 0,006) and multivariate analysis (*p* = 0,010) (Additional file 2: Table S14), it also influenced the E’ wave of the IVSm (*p* = 0,011) (Additional file 2: Table S16), the A’ wave of the IVSb (*p* = 0,015) (Additional file 2: Table S20), and the E’ wave of the IVSb (*p* = 0,028) (Additional file 2: Table S19). HSCT had a significant influence when considering the time from the end of therapy to the evaluation (*p* = 0,024). Anthracyclines equivalent dose was another important risk factor, with significant influence on the E’ wave of the LVPWb (*p* = 0,040) (Additional file 2: Table S14) and on the S wave of the IVSb (*p* = 0,025) (Additional file 2: Table S18). Even the time from the end of therapy to the TDI evaluation had influence on the E’ wave of the IVSb both on linear regression analysis (*p* = 0,024) and multivariate analysis (*p* = 0,020) (Additional file 2: Table S19).

## Discussion

Although clinically-apparent anthracycline-induced heart failure in long-term survivors of childhood cancer ranges between 5 and 20% [13], subclinical cardiomyopathy can be detected by echocardiography in 57% of these patients, with elevated end-systolic wall stress as the most common alteration [1] [14]. Our patients were asymptomatic and had normal systolic function at the time of the evaluation. We measured a significant increment of IVS thickness of the patients compared to the healthy controls and the left ventricular chamber was not dilated. Lipshultz et al. previously observed a dose-dependent reduction in LV wall thickness and increased ventricular dimensions in patients treated with anthracyclines [15], but he considered a median follow-up period of 11.8 years while our cohort median follow-up period was 2 years. Considering that dilated cardiomyopathy is mainly displayed by older patients, the different stage of the follow-up could account for our different results [16]. Our analysis also showed that none of the risk factors evaluated had a significant impact on global systolic function. Previous reports suggested that diastolic dysfunction precedes systolic dysfunction [17]; a delayed ventricular relaxation pattern or a restrictive pattern are typical presentations [18] [19]. Subclinical diastolic dysfunction is also present in survivors of adult-onset malignancy treated with anthracyclines [20]. Our cohort showed statistically significant variations of diastolic-related measures (IVRT, DT, E, A, E’, A’), but they were not specific to any diastolic pattern of dysfunction. E/A ratio and E/E’ ratio were also used as standard measures to assess diastolic function. E, A and E/A ratio values may be subject to “pseudonormalization”, because transmitral inflow Doppler alone is preload-dependent [21]: E/E’ ratio aims to correct this bias, since TDI is not preload-dependent [8]. E/E’ ratio is also considered to be a good predictor of LV filling pressure [22]. Our analysis did not show any statistically significant variation of E/A ratio and E/E’ ratio values between patients and controls. Furthermore, known risk factors of cardiac involvement (such as gender, BSA, chest irradiation, and tumor type) did not influence the diastolic function of the patients in our study. Since the anthracycline-related myocardial dysfunction is a progressive process [16], all of these findings might reflect a very early stage of the cardiac involvement: our cohort median follow-up period is 2 years (against Alehan et al. median 17.5 years [11] and Kapusta et al. median 7,1 years [23]). A prospective evaluation is needed to evaluate the damage progression. Three patients died from the time of the evaluations to the writing of this article; according to the registry of the Italian Association of Pediatric Hematology-Oncology (AIEOP) none of them died of cardiac causes.

**Fig. 2:**
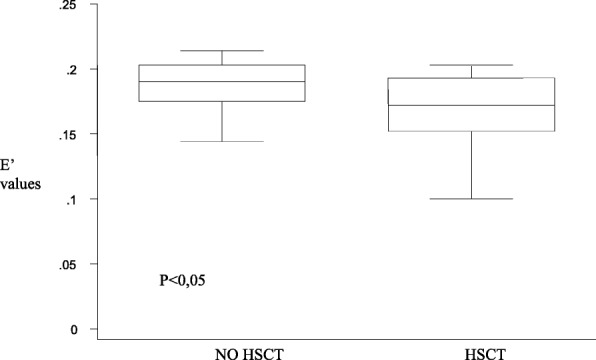
Difference of E’ at basal LVPW between patients who underwent HSCT and those who didn’t

**Fig. 3:**
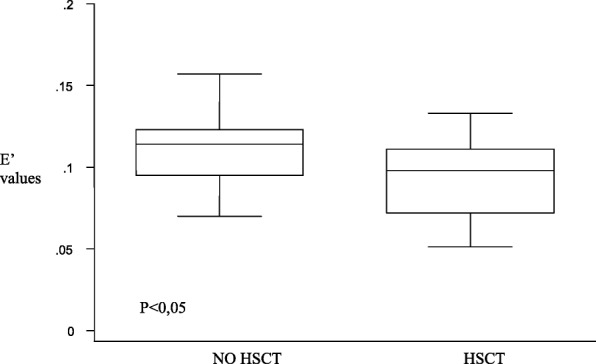
Difference of E’ at middle IVS between patients who underwent HSCT and those who didn’t

**Fig. 4:**
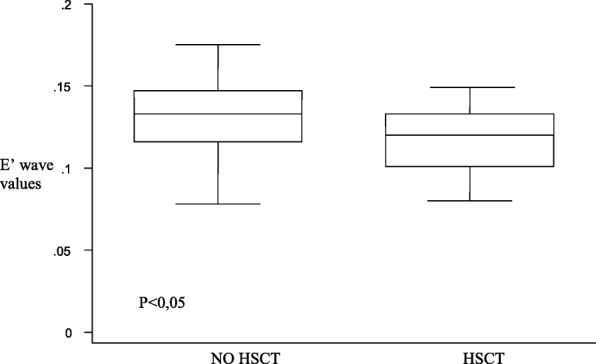
Difference of E’ at basal IVS between patients who underwent HSCT and who didn’t

**Fig. 5:**
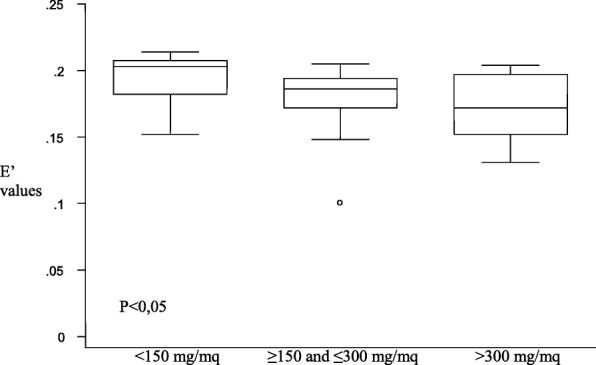
Difference of E’ of basal LVPW between patients who received different total cumulative doses of anthracyclines

**Fig. 6:**
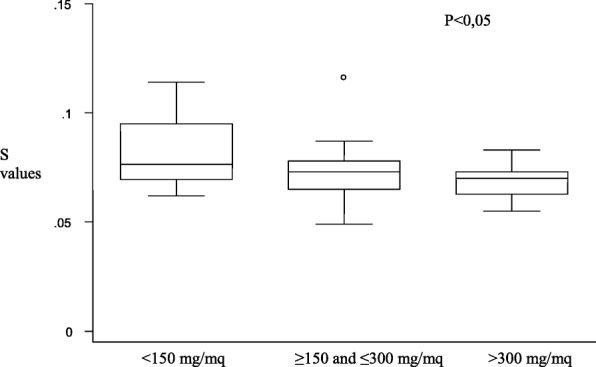
Difference of S of basal IVS between patients who received different total cumulative doses of anthracyclines

**Fig. 7:**
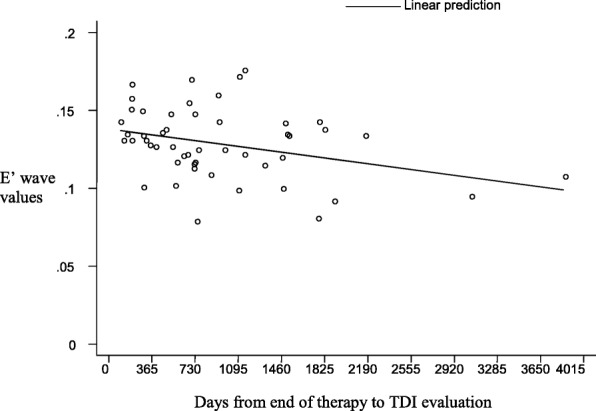
Trend of E’ values of basal IVS after the end of therapy

Patients who underwent HSCT (*n* = 15) had mild alterations on the left ventricular posterior wall (Fig. 2), but they also had both diastolic and systolic involvement of the interventricular septum (Figs. 3 and 4), suggesting that the TDI exploration of the IVS could be useful in the early detection of the myocardial dysfunction in these patients. Total cumulative dose of anthracycline influenced both diastolic (Fig. 5) and systolic (Fig. 6) TDI parameters, confirming the central role of the anthracycline cumulative dose on the myocardial toxicity [1, 4]. HSCT and total cumulative dose of anthracyclines where the two most important factors to influence our measurements. The follow-up duration at the time of evaluation also had a statistical impact on diastolic TDI measurement (Fig. 7).

Overall our data confirmed that cardiac ultrasonography can detect nonspecific diastolic abnormalities even in the first months after the end of therapy; pulsed-wave Tissue Doppler Imaging clearly demonstrated its sensitivity towards these alterations, but it didn’t add any information to the conventional echocardiography at these stages of the follow-up. Karakurt et al. [18] found E/E’ ratio measured at the mitral annulus to be statistically significant in a cohort with similar follow-up times, but the clinical means of these findings is still unclear. Our finding suggested that all children treated with anthracyclines should undergo a regular echocardiographic follow-up as soon as possible after the end of therapy, with at least one conventional ultrasonography in the first year of follow-up (Additional file 1: Table S7A “Addendum”). TDI evaluation is strongly suggested for patients who underwent HSCT based on the results of the linear regression and multivariate analysis. Further evaluations on larger cohorts are needed to confirm these hypothesis.

## Conclusions

Along with secondary malignancies, cardiotoxicity is the leading cause of morbidity and mortality among long-term survivors of childhood cancer [16]. TDI represents a non-invasive, low-cost and sensitive tecnique for the detection of myocardial alterations in childhood cancer survivors. Long-term TDI monitoring has been proven effective in detecting myocardial dysfunctions; TDI monitoring from the early stages of anthracyclines administration could be useful for optimizing the therapy management. Our results showed the sensitivity of the TDI toward mild cardiac impairment but they are not conclusive and need further prospective evaluation.

## Additional files

Additional file 1: Tables of t-test analysis between patient’s cohort subgroups. Due to the large amount of data which would have been impractical to attach to the end of this manuscript, the authors provides this file with eight detailed tables (from “Table 2” to “Table 9”) supporting the results of the analysis. (DOCX 145 kb)

Additional file 2: Tables of linear regression analysis and multivariate analysis. Due to the large amount of data which would have been impractical to attach to the end of this manuscript, the authors provides this file with seventeen detailed tables (from “Table 10” to “Table 26”) supporting the results of the analysis. (DOCX 46 kb)

## Abbreviations

AIEOP: associazione italiana ematologia ed. oncologia pediatrica
BSA: body surface area
DOX: Doxorubicin
DT: E deceleration time
EF: ejection fraction
HSCT: hematopoietic stem cell transplantation
IVRT: left ventricle isovolumic relaxation time
IVSd: interventricular wall septum thickness in diastole
IVSs: interventricular wall septum thickness in systole
LVEDD: left ventricle end-diastolic diameter
LVESD: left ventricle end-systolic diameter
LVIDd: left ventricle internal end-diastolic diameter
LVIDs: left ventricle internal end-systolic diameter
LVPWd: left ventricle posterior wall in diastole
LVPWs: left ventricle posterior wall in systole
MAD: mitral annular displacement
SF: shortening fraction
TDI: Tissue Doppler Imaging
