# Supplementary material for: Tissue Doppler Imaging for anthracycline cardiotoxicity monitoring in pediatric patients with cancer
Source: Cardiooncology. 2018 Sep 3;4:6. doi: 10.1186/s40959-018-0032-3 (PMC7048119; doi:10.1186/s40959-018-0032-3)
Supplement: Supplementary file 1 — Tables of t-test analysis between patient’s cohort subgroups. Due to the large amount of data which would have been impractical to attach to the end of this manuscript, the authors provides this file with eight detailed tables (from “Table 2” to “Table 9”) supporting the results of the analysis. (DOCX 145 kb) [file 40959_2018_32_MOESM1_ESM.docx]

**Table S2: t-test results and Z-scores between different cumulative dose groups.**

|  | **DOSE ≤ 150** | | | | | **DOSE >150** | | | | | **DOSE ≤ 150 Z-score** | | | | |  |
| --- | --- | --- | --- | --- | --- | --- | --- | --- | --- | --- | --- | --- | --- | --- | --- | --- |
|  | mean values (± SD) | | | | | mean values (± SD) | | | | | mean values (± SD) | | | | | **P values** |
|  |  |  |  |  |  |  |  |  |  |  |  |  |  |  |  |  |
| **IVSd** (cm) | 0.861 | ( | ± | 0.173 | ) | 0.863 | ( | ± | 0.137 | ) | 1.43 | ( | ± | 0.91 | ) | 0.9625 |
| **IVSs** (cm) | 1.267 | ( | ± | 0.235 | ) | 1.121 | ( | ± | 0.245 | ) | |  |  |  |  | 0.0830 |
| **LVIDd** (cm) | 3.903 | ( | ± | 0.555 | ) | 4.022 | ( | ± | 0.512 | ) | -1.21 | ( | ± | 1.31 | ) | 0.5078 |
| **LVIDs** (cm) | 2.639 | ( | ± | 0.362 | ) | 2.709 | ( | ± | 0.456 | ) | -0.30 | ( | ± | 0.90 | ) | 0.6413 |
| **LVPWd** (cm) | 0.825 | ( | ± | 0.230 | ) | 0.810 | ( | ± | 0.146 | ) | 0.84 | ( | ± | 1.12 | ) | 0.7940 |
| **LVPWs** (cm) | 1.090 | ( | ± | 0.216 | ) | 1.110 | ( | ± | 0.249 | ) | |  |  |  |  | 0.8029 |
| **FS** (%) | 31.82 | ( | ± | 3.45 | ) | 32.72 | ( | ± | 6.90 | ) | |  |  |  |  | 0.5584 |
| **FE** (%) | 61.60 | ( | ± | 0.1 | ) | 61.85 | ( | ± | 0.11 | ) | |  |  |  |  | 0.9229 |
| **DECEL TIME** (s) | 0.184 | ( | ± | 0.078 | ) | 0.170 | ( | ± | 0.066 | ) | |  |  |  |  | 0.5995 |
| **IVRT** (s) | 0.094 | ( | ± | 0.016 | ) | 0.096 | ( | ± | 0.027 | ) | |  |  |  |  | 0.8692 |
| **E** (m/s) | 0.849 | ( | ± | 0.151 | ) | 0.869 | ( | ± | 0.152 | ) | |  |  |  |  | 0.7123 |
| **A** (m/s) | 0.478 | ( | ± | 0.124 | ) | 0.496 | ( | ± | 0.110 | ) | |  |  |  |  | 0.6666 |
| **E/A RATIO** | 1.85 | ( | ± | 0.43 | ) | 1.83 | ( | ± | 0.43 | ) | |  |  |  |  | 0.9263 |
| *LVPWm* |  |  |  |  |  |  |  |  |  |  |  |  |  |  |  |  |
| **S** (m/s) | 0.111 | ( | ± | 0.04 | ) | 0.092 | ( | ± | 0.02 | ) | |  |  |  |  | 0.1067 |
| **E'** (m/s) | 0.144 | ( | ± | 0.028 | ) | 0.158 | ( | ± | 0.030 | ) | |  |  |  |  | 0.1901 |
| **A'** (m/s) | 0.045 | ( | ± | 0.015 | ) | 0.045 | ( | ± | 0.012 | ) | |  |  |  |  | 0.9885 |
| **E'/A' RATIO** | 3.548 | ( | ± | 1.210 | ) | 3.744 | ( | ± | 1.233 | ) | |  |  |  |  | 0.6411 |
| **E/E' RATIO** | 5.811 | ( | ± | 1.658 | ) | 5.661 | ( | ± | 1.373 | ) | |  |  |  |  | 0.7798 |
| *LVPWb* |  |  |  |  |  |  |  |  |  |  |  |  |  |  |  |  |
| **S** (m/s) | 0.102 | ( | ± | 0.038 | ) | 0.101 | ( | ± | 0.026 | ) | |  |  |  |  | 0.9060 |
| **E'** (m/s) | 0.179 | ( | ± | 0.035 | ) | 0.181 | ( | ± | 0.019 | ) | |  |  |  |  | 0.8484 |
| **A'** (m/s) | 0.056 | ( | ± | 0.022 | ) | 0.053 | ( | ± | 0.016 | ) | |  |  |  |  | 0.6978 |
| **E'/A' RATIO** | 3.638 | ( | ± | 1.293 | ) | 3.681 | ( | ± | 1.028 | ) | |  |  |  |  | 0.9086 |
| **E/E' RATIO** | 4.621 | ( | ± | 0.860 | ) | 4.832 | ( | ± | 1.018 | ) | |  |  |  |  | 0.5703 |
| *IVSm* |  |  |  |  |  |  |  |  |  |  |  |  |  |  |  |  |
| **S** (m/s) | 0.066 | ( | ± | 0.016 | ) | 0.057 | ( | ± | 0.007 | ) | |  |  |  |  | 0.1565 |
| **E'** (m/s) | 0.100 | ( | ± | 0.018 | ) | 0.108 | ( | ± | 0.025 | ) | |  |  |  |  | 0.3208 |
| **A'** (m/s) | 0.043 | ( | ± | 0.012 | ) | 0.041 | ( | ± | 0.012 | ) | |  |  |  |  | 0.5476 |
| **E'/A' RATIO** | 2.443 | ( | ± | 0.719 | ) | 2.779 | ( | ± | 0.894 | ) | |  |  |  |  | 0.2582 |
| **E/E' RATIO** | 8.432 | ( | ± | 2.300 | ) | 8.518 | ( | ± | 2.606 | ) | |  |  |  |  | 0.9278 |
| *IVSb* |  |  |  |  |  |  |  |  |  |  |  |  |  |  |  |  |
| **S** (m/s) | 0.086 | ( | ± | 0.020 | ) | 0.071 | ( | ± | 0.009 | ) | |  |  |  |  | 0.0682 |
| **E'** (m/s) | 0.131 | ( | ± | 0.017 | ) | 0.127 | ( | ± | 0.024 | ) | |  |  |  |  | 0.6241 |
| **A'** (m/s) | 0.049 | ( | ± | 0.020 | ) | 0.048 | ( | ± | 0.010 | ) | |  |  |  |  | 0.8537 |
| **E'/A' RATIO** | 2.959 | ( | ± | 0.994 | ) | 2.794 | ( | ± | 0.932 | ) | |  |  |  |  | 0.6108 |
| **E/E' RATIO** | 6.341 | ( | ± | 1.219 | ) | 7.052 | ( | ± | 2.001 | ) | |  |  |  |  | 0.3152 |
| *MAD* |  |  |  |  |  |  |  |  |  |  |  |  |  |  |  |  |
| **S** (m/s) | 0.084 | ( | ± | 0.026 | ) | 0.076 | ( | ± | 0.020 | ) | |  |  |  |  | 0.5523 |
| **E'** (m/s) | 0.141 | ( | ± | 0.035 | ) | 0.146 | ( | ± | 0.030 | ) | |  |  |  |  | 0.6948 |
| **A'** (m/s) | 0.051 | ( | ± | 0.020 | ) | 0.050 | ( | ± | 0.020 | ) | |  |  |  |  | 0.9094 |
| **E'/A' RATIO** | 3.461 | ( | ± | 2.348 | ) | 3.388 | ( | ± | 1.549 | ) | |  |  |  |  | 0.9046 |
| **E/E' RATIO** | 6.063 | ( | ± | 2.551 | ) | 6.101 | ( | ± | 1.608 | ) | |  |  |  |  | 0.9551 |

MAD: mitral annular displacement; LVPWm middle left ventricular posterior wall; LVPWb: basal left ventricular posterior wall; IVSm: middle interventricular septum; IVSb: basal interventricular septum.

**Table S3: t-test results and Z-scores between different cumulative dose groups.**

|  | **DOSE <=300** | | | | | **DOSE >300** | | | | | **DOSE >300 Z-score** | | | | |  |
| --- | --- | --- | --- | --- | --- | --- | --- | --- | --- | --- | --- | --- | --- | --- | --- | --- |
|  | mean values (± SD) | | | | | mean values (± SD) | | | | | mean values (± SD) | | | | | **P values** |
|  |  |  |  |  |  |  |  |  |  |  |  |  |  |  |  |  |
| **IVSd** (cm) | 0.878 | ( | ± | 0.148 | ) | 0.827 | ( | ± | 0.130 | ) | 1.85 | ( | ± | 1.24 | ) | 0.2472 |
| **IVSs** (cm) | 1.184 | ( | ± | 0.266 | ) | 1.079 | ( | ± | 0.189 | ) | |  |  |  |  | 0.1725 |
| **LVIDd** (cm) | 3.951 | ( | ± | 0.513 | ) | 4.099 | ( | ± | 0.534 | ) | -0.08 | ( | ± | 1.21 | ) | 0.3588 |
| **LVIDs** (cm) | 2.652 | ( | ± | 0.427 | ) | 2.791 | ( | ± | 0.452 | ) | 0.45 | ( | ± | 1.21 | ) | 0.3038 |
| **LVPWd** (cm) | 0.817 | ( | ± | 0.174 | ) | 0.805 | ( | ± | 0.148 | ) | 1.11 | ( | ± | 1.12 | ) | 0.8302 |
| **LVPWs** (cm) | 1.091 | ( | ± | 0.254 | ) | 1.139 | ( | ± | 0.208 | ) | |  |  |  |  | 0.5240 |
| **FS** (%) | 32.97 | ( | ± | 6.78 | ) | 31.45 | ( | ± | 4.99 | ) | |  |  |  |  | 0.4386 |
| **FE** (%) | 62.07 | ( | ± | 0.1 | ) | 61.15 | ( | ± | 0.08 | ) | |  |  |  |  | 0.7721 |
| **DECEL TIME** (s) | 0.168 | ( | ± | 0.066 | ) | 0.184 | ( | ± | 0.075 | ) | |  |  |  |  | 0.4997 |
| **IVRT** (s) | 0.094 | ( | ± | 0.022 | ) | 0.098 | ( | ± | 0.030 | ) | |  |  |  |  | 0.6659 |
| **E** (m/s) | 0.855 | ( | ± | 0.160 | ) | 0.886 | ( | ± | 0.131 | ) | |  |  |  |  | 0.5183 |
| **A** (m/s) | 0.500 | ( | ± | 0.114 | ) | 0.476 | ( | ± | 0.109 | ) | |  |  |  |  | 0.4960 |
| **E/A RATIO** | 1.79 | ( | ± | 0.40 | ) | 1.93 | ( | ± | 0.46 | ) | |  |  |  |  | 0.3117 |
| *LVPWm* |  |  |  |  |  |  |  |  |  |  |  |  |  |  |  |  |
| **S** (m/s) | 0.097 | ( | ± | 0.03 | ) | 0.093 | ( | ± | 0.03 | ) | |  |  |  |  | 0.6938 |
| **E'** (m/s) | 0.155 | ( | ± | 0.029 | ) | 0.153 | ( | ± | 0.034 | ) | |  |  |  |  | 0.7964 |
| **A'** (m/s) | 0.046 | ( | ± | 0.013 | ) | 0.043 | ( | ± | 0.013 | ) | |  |  |  |  | 0.4231 |
| **E'/A' RATIO** | 3.623 | ( | ± | 1.146 | ) | 3.882 | ( | ± | 1.399 | ) | |  |  |  |  | 0.4976 |
| **E/E' RATIO** | 5.523 | ( | ± | 1.337 | ) | 6.036 | ( | ± | 1.552 | ) | |  |  |  |  | 0.2529 |
| *LVPWb* |  |  |  |  |  |  |  |  |  |  |  |  |  |  |  |  |
| **S** (m/s) | 0.100 | ( | ± | 0.028 | ) | 0.105 | ( | ± | 0.030 | ) | |  |  |  |  | 0.4928 |
| **E'** (m/s) | 0.184 | ( | ± | 0.022 | ) | 0.173 | ( | ± | 0.024 | ) | |  |  |  |  | 0.1244 |
| **A'** (m/s) | 0.055 | ( | ± | 0.019 | ) | 0.051 | ( | ± | 0.013 | ) | |  |  |  |  | 0.4527 |
| **E'/A' RATIO** | 3.702 | ( | ± | 1.152 | ) | 3.599 | ( | ± | 0.912 | ) | |  |  |  |  | 0.7601 |
| **E/E' RATIO** | 4.588 | ( | ± | 0.858 | ) | 5.210 | ( | ± | 1.121 | ) | |  |  |  |  | 0.0433 |
| *IVSm* |  |  |  |  |  |  |  |  |  |  |  |  |  |  |  |  |
| **S** (m/s) | 0.060 | ( | ± | 0.011 | ) | 0.057 | ( | ± | 0.008 | ) | |  |  |  |  | 0.8382 |
| **E'** (m/s) | 0.110 | ( | ± | 0.022 | ) | 0.097 | ( | ± | 0.027 | ) | |  |  |  |  | 0.0934 |
| **A'** (m/s) | 0.043 | ( | ± | 0.013 | ) | 0.038 | ( | ± | 0.007 | ) | |  |  |  |  | 0.0839 |
| **E'/A' RATIO** | 2.739 | ( | ± | 0.872 | ) | 2.625 | ( | ± | 0.868 | ) | |  |  |  |  | 0.6738 |
| **E/E' RATIO** | 7.889 | ( | ± | 1.870 | ) | 9.767 | ( | ± | 3.233 | ) | |  |  |  |  | 0.0508 |
| *IVSb* |  |  |  |  |  |  |  |  |  |  |  |  |  |  |  |  |
| **S** (m/s) | 0.076 | ( | ± | 0.015 | ) | 0.069 | ( | ± | 0.008 | ) | |  |  |  |  | 0.0616 |
| **E'** (m/s) | 0.131 | ( | ± | 0.021 | ) | 0.121 | ( | ± | 0.026 | ) | |  |  |  |  | 0.1490 |
| **A'** (m/s) | 0.048 | ( | ± | 0.014 | ) | 0.050 | ( | ± | 0.010 | ) | |  |  |  |  | 0.7092 |
| **E'/A' RATIO** | 2.940 | ( | ± | 0.939 | ) | 2.574 | ( | ± | 0.916 | ) | |  |  |  |  | 0.2095 |
| **E/E' RATIO** | 6.529 | ( | ± | 1.367 | ) | 7.707 | ( | ± | 2.530 | ) | |  |  |  |  | 0.1085 |
| *MAD* |  |  |  |  |  |  |  |  |  |  |  |  |  |  |  |  |
| **S** (m/s) | 0.078 | ( | ± | 0.024 | ) | 0.077 | ( | ± | 0.012 | ) | |  |  |  |  | 0.9282 |
| **E'** (m/s) | 0.147 | ( | ± | 0.029 | ) | 0.140 | ( | ± | 0.035 | ) | |  |  |  |  | 0.4808 |
| **A'** (m/s) | 0.051 | ( | ± | 0.022 | ) | 0.047 | ( | ± | 0.010 | ) | |  |  |  |  | 0.3934 |
| **E'/A' RATIO** | 3.535 | ( | ± | 1.938 | ) | 3.079 | ( | ± | 1.042 | ) | |  |  |  |  | 0.2953 |
| **E/E' RATIO** | 5.925 | ( | ± | 1.976 | ) | 6.468 | ( | ± | 1.319 | ) | |  |  |  |  | 0.3550 |

MAD: mitral annular displacement; LVPWm middle left ventricular posterior wall; LVPWb: basal left ventricular posterior wall; IVSm: middle interventricular septum; IVSb: basal interventricular septum.

**Table S4: t-test results and Z-scores between sex groups.**

|  | **FEMALE** | | | | | **MALE** | | | | | **FEMALE Z-score** | | | | |  |
| --- | --- | --- | --- | --- | --- | --- | --- | --- | --- | --- | --- | --- | --- | --- | --- | --- |
|  | mean values (± SD) | | | | | mean values (± SD) | | | | | mean values (± SD) | | | | | **P values** |
|  |  |  |  |  |  |  |  |  |  |  |  |  |  |  |  |  |
| **IVSd** (cm) | 0.843 | ( | ± | 0.139 | ) | 0.874 | ( | ± | 0.147 | ) | 2.15 | ( | ± | 1.08 | ) | 0.4596 |
| **IVSs** (cm) | 1.120 | ( | ± | 0.163 | ) | 1.171 | ( | ± | 0.286 | ) | |  |  |  |  | 0.4212 |
| **LVIDd** (cm) | 3.901 | ( | ± | 0.360 | ) | 4.049 | ( | ± | 0.588 | ) | -0.60 | ( | ± | 0.79 | ) | 0.2749 |
| **LVIDs** (cm) | 2.610 | ( | ± | 0.327 | ) | 2.741 | ( | ± | 0.483 | ) | 0.01 | ( | ± | 0.95 | ) | 0.3115 |
| **LVPWd** (cm) | 0.768 | ( | ± | 0.155 | ) | 0.838 | ( | ± | 0.168 | ) | 0.82 | ( | ± | 0.96 | ) | 0.1526 |
| **LVPWs** (cm) | 1.072 | ( | ± | 0.249 | ) | 1.125 | ( | ± | 0.237 | ) | |  |  |  |  | 0.4545 |
| **FS** (%) | 33.17 | ( | ± | 4.63 | ) | 32.15 | ( | ± | 7.09 | ) | |  |  |  |  | 0.5859 |
| **FE** (%) | 62.16 | ( | ± | 0.1 | ) | 61.58 | ( | ± | 0.12 | ) | |  |  |  |  | 0.8230 |
| **DECEL TIME** (s) | 0.159 | ( | ± | 0.072 | ) | 0.183 | ( | ± | 0.066 | ) | |  |  |  |  | 0.2804 |
| **IVRT** (s) | 0.090 | ( | ± | 0.027 | ) | 0.099 | ( | ± | 0.023 | ) | |  |  |  |  | 0.2354 |
| **E** (m/s) | 0.844 | ( | ± | 0.139 | ) | 0.879 | ( | ± | 0.158 | ) | |  |  |  |  | 0.4356 |
| **A** (m/s) | 0.497 | ( | ± | 0.110 | ) | 0.490 | ( | ± | 0.114 | ) | |  |  |  |  | 0.8199 |
| **E/A RATIO** | 1.75 | ( | ± | 0.38 | ) | 1.89 | ( | ± | 0.45 | ) | |  |  |  |  | 0.2663 |
| *LVPWm* |  |  |  |  |  |  |  |  |  |  |  |  |  |  |  |  |
| **S** (m/s) | 0.090 | ( | ± | 0.03 | ) | 0.099 | ( | ± | 0.03 | ) | |  |  |  |  | 0.2921 |
| **E'** (m/s) | 0.159 | ( | ± | 0.028 | ) | 0.152 | ( | ± | 0.031 | ) | |  |  |  |  | 0.4022 |
| **A'** (m/s) | 0.043 | ( | ± | 0.007 | ) | 0.046 | ( | ± | 0.015 | ) | |  |  |  |  | 0.2564 |
| **E'/A' RATIO** | 3.856 | ( | ± | 1.041 | ) | 3.614 | ( | ± | 1.316 | ) | |  |  |  |  | 0.5050 |
| **E/E' RATIO** | 5.389 | ( | ± | 1.008 | ) | 5.885 | ( | ± | 1.611 | ) | |  |  |  |  | 0.2057 |
| *LVPWb* |  |  |  |  |  |  |  |  |  |  |  |  |  |  |  |  |
| **S** (m/s) | 0.101 | ( | ± | 0.023 | ) | 0.102 | ( | ± | 0.031 | ) | |  |  |  |  | 0.7357 |
| **E'** (m/s) | 0.183 | ( | ± | 0.022 | ) | 0.180 | ( | ± | 0.023 | ) | |  |  |  |  | 0.6377 |
| **A'** (m/s) | 0.055 | ( | ± | 0.017 | ) | 0.053 | ( | ± | 0.018 | ) | |  |  |  |  | 0.6477 |
| **E'/A' RATIO** | 3.512 | ( | ± | 0.846 | ) | 3.761 | ( | ± | 1.192 | ) | |  |  |  |  | 0.4375 |
| **E/E' RATIO** | 4.644 | ( | ± | 0.764 | ) | 4.885 | ( | ± | 1.106 | ) | |  |  |  |  | 0.4227 |
| *IVSm* |  |  |  |  |  |  |  |  |  |  |  |  |  |  |  |  |
| **S** (m/s) | 0.059 | ( | ± | 0.010 | ) | 0.059 | ( | ± | 0.010 | ) | |  |  |  |  | 0.8592 |
| **E'** (m/s) | 0.105 | ( | ± | 0.022 | ) | 0.106 | ( | ± | 0.025 | ) | |  |  |  |  | 0.8651 |
| **A'** (m/s) | 0.044 | ( | ± | 0.015 | ) | 0.040 | ( | ± | 0.009 | ) | |  |  |  |  | 0.2649 |
| **E'/A' RATIO** | 2.545 | ( | ± | 0.805 | ) | 2.795 | ( | ± | 0.894 | ) | |  |  |  |  | 0.3325 |
| **E/E' RATIO** | 8.340 | ( | ± | 2.210 | ) | 8.604 | ( | ± | 2.743 | ) | |  |  |  |  | 0.7333 |
| *IVSb* |  |  |  |  |  |  |  |  |  |  |  |  |  |  |  |  |
| **S** (m/s) | 0.076 | ( | ± | 0.015 | ) | 0.073 | ( | ± | 0.013 | ) | |  |  |  |  | 0.4984 |
| **E'** (m/s) | 0.136 | ( | ± | 0.020 | ) | 0.124 | ( | ± | 0.023 | ) | |  |  |  |  | 0.0762 |
| **A'** (m/s) | 0.051 | ( | ± | 0.016 | ) | 0.047 | ( | ± | 0.011 | ) | |  |  |  |  | 0.2751 |
| **E'/A' RATIO** | 2.860 | ( | ± | 0.917 | ) | 2.814 | ( | ± | 0.964 | ) | |  |  |  |  | 0.8696 |
| **E/E' RATIO** | 6.308 | ( | ± | 1.218 | ) | 7.302 | ( | ± | 2.140 | ) | |  |  |  |  | 0.0513 |
| *MAD* |  |  |  |  |  |  |  |  |  |  |  |  |  |  |  |  |
| **S** (m/s) | 0.072 | ( | ± | 0.014 | ) | 0.081 | ( | ± | 0.024 | ) | |  |  |  |  | 0.1087 |
| **E'** (m/s) | 0.148 | ( | ± | 0.031 | ) | 0.142 | ( | ± | 0.031 | ) | |  |  |  |  | 0.5122 |
| **A'** (m/s) | 0.053 | ( | ± | 0.021 | ) | 0.049 | ( | ± | 0.019 | ) | |  |  |  |  | 0.5340 |
| **E'/A' RATIO** | 3.368 | ( | ± | 1.914 | ) | 3.426 | ( | ± | 1.649 | ) | |  |  |  |  | 0.9126 |
| **E/E' RATIO** | 5.937 | ( | ± | 1.581 | ) | 6.198 | ( | ± | 1.955 | ) | |  |  |  |  | 0.6394 |

MAD: mitral annular displacement; LVPWm middle left ventricular posterior wall; LVPWb: basal left ventricular posterior wall; IVSm: middle interventricular septum; IVSb: basal interventricular septum.

**Table S5: t-test results and Z-scores between different X-rays exposure groups.**

|  | **NOT IRRADIATED** | | | | | **IRRADIATED** | | | | | **IRRAD Z-score** | | | | |  |
| --- | --- | --- | --- | --- | --- | --- | --- | --- | --- | --- | --- | --- | --- | --- | --- | --- |
|  | mean values (± SD) | | | | | mean values (± SD) | | | | | mean values (± SD) | | | | | **P values** |
|  |  |  |  |  |  |  |  |  |  |  |  |  |  |  |  |  |
| **IVSd** (cm) | 0.863 | ( | ± | 0.140 | ) | 0.864 | ( | ± | 0.165 | ) | 1.84 | ( | ± | 1.21 | ) | 0.9704 |
| **IVSs** (cm) | 1.139 | ( | ± | 0.260 | ) | 1.201 | ( | ± | 0.203 | ) | |  |  |  |  | 0.4706 |
| **LVIDd** (cm) | 4.016 | ( | ± | 0.552 | ) | 3.923 | ( | ± | 0.388 | ) | -1.03 | ( | ± | 1.27 | ) | 0.6038 |
| **LVIDs** (cm) | 2.705 | ( | ± | 0.459 | ) | 2.654 | ( | ± | 0.353 | ) | -0.18 | ( | ± | 1.09 | ) | 0.7325 |
| **LVPWd** (cm) | 0.808 | ( | ± | 0.168 | ) | 0.833 | ( | ± | 0.161 | ) | 1.17 | ( | ± | 1.04 | ) | 0.6567 |
| **LVPWs** (cm) | 1.110 | ( | ± | 0.240 | ) | 1.091 | ( | ± | 0.251 | ) | |  |  |  |  | 0.8224 |
| **FS** (%) | 32.64 | ( | ± | 6.64 | ) | 32.09 | ( | ± | 5.05 | ) | |  |  |  |  | 0.8016 |
| **FE** (%) | 61.56 | ( | ± | 0.1 | ) | 62.63 | ( | ± | 0.09 | ) | |  |  |  |  | 0.7586 |
| **DECEL TIME** (s) | 0.173 | ( | ± | 0.072 | ) | 0.172 | ( | ± | 0.060 | ) | |  |  |  |  | 0.9657 |
| **IVRT** (s) | 0.096 | ( | ± | 0.026 | ) | 0.094 | ( | ± | 0.020 | ) | |  |  |  |  | 0.8113 |
| **E** (m/s) | 0.842 | ( | ± | 0.143 | ) | 0.941 | ( | ± | 0.154 | ) | |  |  |  |  | 0.5560 |
| **A** (m/s) | 0.472 | ( | ± | 0.107 | ) | 0.558 | ( | ± | 0.105 | ) | |  |  |  |  | 0.0239 |
| **E/A RATIO** | 1.87 | ( | ± | 0.41 | ) | 1.73 | ( | ± | 0.47 | ) | |  |  |  |  | 0.3140 |
| *LVPWm* |  |  |  |  |  |  |  |  |  |  |  |  |  |  |  |  |
| **S** (m/s) | 0.097 | ( | ± | 0.02 | ) | 0.092 | ( | ± | 0.03 | ) | |  |  |  |  | 0.6417 |
| **E'** (m/s) | 0.154 | ( | ± | 0.033 | ) | 0.157 | ( | ± | 0.018 | ) | |  |  |  |  | 0.7346 |
| **A'** (m/s) | 0.044 | ( | ± | 0.010 | ) | 0.050 | ( | ± | 0.019 | ) | |  |  |  |  | 0.2998 |
| **E'/A' RATIO** | 3.720 | ( | ± | 1.133 | ) | 3.633 | ( | ± | 1.546 | ) | |  |  |  |  | 0.8367 |
| **E/E' RATIO** | 5.556 | ( | ± | 1.381 | ) | 6.118 | ( | ± | 1.502 | ) | |  |  |  |  | 0.2547 |
| *LVPWb* |  |  |  |  |  |  |  |  |  |  |  |  |  |  |  |  |
| **S** (m/s) | 0.100 | ( | ± | 0.027 | ) | 0.105 | ( | ± | 0.033 | ) | |  |  |  |  | 0.5692 |
| **E'** (m/s) | 0.180 | ( | ± | 0.024 | ) | 0.183 | ( | ± | 0.020 | ) | |  |  |  |  | 0.7815 |
| **A'** (m/s) | 0.052 | ( | ± | 0.014 | ) | 0.060 | ( | ± | 0.027 | ) | |  |  |  |  | 0.3713 |
| **E'/A' RATIO** | 3.689 | ( | ± | 0.968 | ) | 3.610 | ( | ± | 1.459 | ) | |  |  |  |  | 0.8341 |
| **E/E' RATIO** | 4.647 | ( | ± | 0.843 | ) | 5.249 | ( | ± | 1.279 | ) | |  |  |  |  | 0.0763 |
| *IVSm* |  |  |  |  |  |  |  |  |  |  |  |  |  |  |  |  |
| **S** (m/s) | 0.060 | ( | ± | 0.010 | ) | 0.055 | ( | ± | 0.010 | ) | |  |  |  |  | 0.1706 |
| **E'** (m/s) | 0.105 | ( | ± | 0.021 | ) | 0.109 | ( | ± | 0.031 | ) | |  |  |  |  | 0.6516 |
| **A'** (m/s) | 0.042 | ( | ± | 0.012 | ) | 0.038 | ( | ± | 0.009 | ) | |  |  |  |  | 0.2855 |
| **E'/A' RATIO** | 2.639 | ( | ± | 0.831 | ) | 2.937 | ( | ± | 0.977 | ) | |  |  |  |  | 0.3170 |
| **E/E' RATIO** | 8.189 | ( | ± | 1.977 | ) | 9.494 | ( | ± | 3.740 | ) | |  |  |  |  | 0.2894 |
| *IVSb* |  |  |  |  |  |  |  |  |  |  |  |  |  |  |  |  |
| **S** (m/s) | 0.075 | ( | ± | 0.015 | ) | 0.070 | ( | ± | 0.009 | ) | |  |  |  |  | 0.2469 |
| **E'** (m/s) | 0.128 | ( | ± | 0.023 | ) | 0.129 | ( | ± | 0.022 | ) | |  |  |  |  | 0.8710 |
| **A'** (m/s) | 0.049 | ( | ± | 0.013 | ) | 0.048 | ( | ± | 0.011 | ) | |  |  |  |  | 0.7850 |
| **E'/A' RATIO** | 2.823 | ( | ± | 0.965 | ) | 2.854 | ( | ± | 0.878 | ) | |  |  |  |  | 0.9247 |
| **E/E'** | 6.703 | ( | ± | 1.606 | ) | 7.581 | ( | ± | 2.560 | ) | |  |  |  |  | 0.1804 |
| *MAD* |  |  |  |  |  |  |  |  |  |  |  |  |  |  |  |  |
| **S** (m/s) | 0.078 | ( | ± | 0.023 | ) | 0.074 | ( | ± | 0.012 | ) | |  |  |  |  | 0.5409 |
| **E'** (m/s) | 0.142 | ( | ± | 0.032 | ) | 0.157 | ( | ± | 0.025 | ) | |  |  |  |  | 0.1699 |
| **A'** (m/s) | 0.050 | ( | ± | 0.020 | ) | 0.052 | ( | ± | 0.021 | ) | |  |  |  |  | 0.7300 |
| **E'/A' RATIO** | 3.355 | ( | ± | 1.716 | ) | 3.598 | ( | ± | 1.871 | ) | |  |  |  |  | 0.6966 |
| **E/E' RATIO** | 6.130 | ( | ± | 1.952 | ) | 5.968 | ( | ± | 1.192 | ) | |  |  |  |  | 0.8059 |

MAD: mitral annular displacement; LVPWm middle left ventricular posterior wall; LVPWb: basal left ventricular posterior wall; IVSm: middle interventricular septum; IVSb: basal interventricular septum.

**Table S6: t-test results and Z-scores between patients who underwent HSCT and those who didn’t.**

|  | **NO HSCT** | | | | | **HSCT** | | | | | **HSCT Z-score** | | | | |  |
| --- | --- | --- | --- | --- | --- | --- | --- | --- | --- | --- | --- | --- | --- | --- | --- | --- |
|  | mean values (± SD) | | | | | mean values (± SD) | | | | | mean values (± SD) | | | | | **P values** |
|  |  |  |  |  |  |  |  |  |  |  |  |  |  |  |  |  |
| **IVSd** (cm) | 0.879 | ( | ± | 0.143 | ) | 0.825 | ( | ± | 0.143 | ) | 1.94 | ( | ± | 1.20 | ) | 0.2292 |
| **IVSs** (cm) | 1.172 | ( | ± | 0.283 | ) | 1.108 | ( | ± | 0.136 | ) | |  |  |  |  | 0.2864 |
| **LVIDd** (cm) | 4.026 | ( | ± | 0.511 | ) | 3.924 | ( | ± | 0.547 | ) | -0.42 | ( | ± | 1.44 | ) | 0.5294 |
| **LVIDs** (cm) | 2.709 | ( | ± | 0.441 | ) | 2.658 | ( | ± | 0.432 | ) | 0.27 | ( | ± | 1.35 | ) | 0.7072 |
| **LVPWd** (cm) | 0.809 | ( | ± | 0.170 | ) | 0.823 | ( | ± | 0.160 | ) | 1.34 | ( | ± | 1.06 | ) | 0.7977 |
| **LVPWs** (cm) | 1.109 | ( | ± | 0.239 | ) | 1.099 | ( | ± | 0.252 | ) | |  |  |  |  | 0.8907 |
| **FS** (%) | 32.88 | ( | ± | 6.71 | ) | 31.67 | ( | ± | 5.27 | ) | |  |  |  |  | 0.5393 |
| **FE** (%) | 61.87 | ( | ± | 0.1 | ) | 61.60 | ( | ± | 0.08 | ) | |  |  |  |  | 0.9306 |
| **DECEL TIME** (s) | 0.179 | ( | ± | 0.069 | ) | 0.162 | ( | ± | 0.069 | ) | |  |  |  |  | 0.4817 |
| **IVRT** (s) | 0.096 | ( | ± | 0.022 | ) | 0.095 | ( | ± | 0.030 | ) | |  |  |  |  | 0.8980 |
| **E** (m/s) | 0.859 | ( | ± | 0.153 | ) | 0.881 | ( | ± | 0.149 | ) | |  |  |  |  | 0.6495 |
| **A** (m/s) | 0.490 | ( | ± | 0.113 | ) | 0.498 | ( | ± | 0.112 | ) | |  |  |  |  | 0.8348 |
| **E/A RATIO** | 1.84 | ( | ± | 0.40 | ) | 1.83 | ( | ± | 0.49 | ) | |  |  |  |  | 0.9198 |
| *LVPWm* |  |  |  |  |  |  |  |  |  |  |  |  |  |  |  |  |
| **S** (m/s) | 0.096 | ( | ± | 0.02 | ) | 0.096 | ( | ± | 0.03 | ) | |  |  |  |  | 0.8943 |
| **E'** (m/s) | 0.156 | ( | ± | 0.029 | ) | 0.152 | ( | ± | 0.034 | ) | |  |  |  |  | 0.7104 |
| **A'** (m/s) | 0.044 | ( | ± | 0.010 | ) | 0.047 | ( | ± | 0.018 | ) | |  |  |  |  | 0.6443 |
| **E'/A' RATIO** | 3.652 | ( | ± | 0.921 | ) | 3.816 | ( | ± | 1.765 | ) | |  |  |  |  | 0.7366 |
| **E/E' RATIO** | 5.637 | ( | ± | 1.308 | ) | 5.813 | ( | ± | 1.680 | ) | |  |  |  |  | 0.7026 |
| *LVPWb* |  |  |  |  |  |  |  |  |  |  |  |  |  |  |  |  |
| **S** (m/s) | 0.101 | ( | ± | 0.028 | ) | 0.102 | ( | ± | 0.029 | ) | |  |  |  |  | 0.7428 |
| **E'** (m/s) | 0.186 | ( | ± | 0.017 | ) | 0.168 | ( | ± | 0.029 | ) | |  |  |  |  | 0.0303 |
| **A'** (m/s) | 0.052 | ( | ± | 0.016 | ) | 0.057 | ( | ± | 0.021 | ) | |  |  |  |  | 0.3537 |
| **E'/A' RATIO** | 3.827 | ( | ± | 0.976 | ) | 3.308 | ( | ± | 1.245 | ) | |  |  |  |  | 0.1198 |
| **E/E' RATIO** | 4.618 | ( | ± | 0.851 | ) | 5.186 | ( | ± | 1.176 | ) | |  |  |  |  | 0.0712 |
| *IVSm* |  |  |  |  |  |  |  |  |  |  |  |  |  |  |  |  |
| **S** (m/s) | 0.058 | ( | ± | 0.010 | ) | 0.060 | ( | ± | 0.010 | ) | |  |  |  |  | 0.8031 |
| **E'** (m/s) | 0.112 | ( | ± | 0.021 | ) | 0.093 | ( | ± | 0.025 | ) | |  |  |  |  | 0.0109 |
| **A'** (m/s) | 0.040 | ( | ± | 0.012 | ) | 0.044 | ( | ± | 0.011 | ) | |  |  |  |  | 0.2730 |
| **E'/A' RATIO** | 2.930 | ( | ± | 0.858 | ) | 2.179 | ( | ± | 0.630 | ) | |  |  |  |  | 0.0037 |
| **E/E' RATIO** | 7.872 | ( | ± | 1.714 | ) | 9.939 | ( | ± | 3.444 | ) | |  |  |  |  | 0.0488 |
| *IVSb* |  |  |  |  |  |  |  |  |  |  |  |  |  |  |  |  |
| **S** (m/s) | 0.072 | ( | ± | 0.010 | ) | 0.077 | ( | ± | 0.018 | ) | |  |  |  |  | 0.2788 |
| **E'** (m/s) | 0.133 | ( | ± | 0.023 | ) | 0.118 | ( | ± | 0.019 | ) | |  |  |  |  | 0.0282 |
| **A'** (m/s) | 0.046 | ( | ± | 0.011 | ) | 0.055 | ( | ± | 0.015 | ) | |  |  |  |  | 0.0151 |
| **E'/A' RATIO** | 3.091 | ( | ± | 0.963 | ) | 2.223 | ( | ± | 0.512 | ) | |  |  |  |  | 0.0001 |
| **E/E' RATIO** | 6.571 | ( | ± | 1.500 | ) | 7.695 | ( | ± | 2.444 | ) | |  |  |  |  | 0.1290 |
| *MAD* |  |  |  |  |  |  |  |  |  |  |  |  |  |  |  |  |
| **S** (m/s) | 0.075 | ( | ± | 0.016 | ) | 0.083 | ( | ± | 0.029 | ) | |  |  |  |  | 0.2775 |
| **E'** (m/s) | 0.148 | ( | ± | 0.029 | ) | 0.136 | ( | ± | 0.035 | ) | |  |  |  |  | 0.2150 |
| **A'** (m/s) | 0.051 | ( | ± | 0.019 | ) | 0.049 | ( | ± | 0.022 | ) | |  |  |  |  | 0.8526 |
| **E'/A' RATIO** | 3.325 | ( | ± | 1.379 | ) | 3.604 | ( | ± | 2.457 | ) | |  |  |  |  | 0.6941 |
| **E/E' RATIO** | 5.907 | ( | ± | 1.719 | ) | 6.552 | ( | ± | 1.982 | ) | |  |  |  |  | 0.2812 |

MAD: mitral annular displacement; LVPWm middle left ventricular posterior wall; LVPWb: basal left ventricular posterior wall; IVSm: middle interventricular septum; IVSb: basal interventricular septum.

**Table S7: t-test results and Z-scores between tumor type groups.**

|  | **LEUKEMIA** | | | | | **SOLID TUMOR** | | | | | **LEUKEMIA Z-score** | | | | |  |
| --- | --- | --- | --- | --- | --- | --- | --- | --- | --- | --- | --- | --- | --- | --- | --- | --- |
|  | mean values (± SD) | | | | | mean values (± SD) | | | | | mean values (± SD) | | | | | **P values** |
|  |  |  |  |  |  |  |  |  |  |  |  |  |  |  |  |  |
| **IVSd** (cm) | 0.867 | ( | ± | 0.151 | ) | 0.856 | ( | ± | 0.136 | ) | 2.30 | ( | ± | 1.49 | ) | 0.8007 |
| **IVSs** (cm) | 1.131 | ( | ± | 0.283 | ) | 1.188 | ( | ± | 0.178 | ) | |  |  |  |  | 0.3870 |
| **LVIDd** (cm) | 3.998 | ( | ± | 0.554 | ) | 3.991 | ( | ± | 0.469 | ) | -0.39 | ( | ± | 1.42 | ) | 0.9636 |
| **LVIDs** (cm) | 2.703 | ( | ± | 0.489 | ) | 2.678 | ( | ± | 0.340 | ) | 0.24 | ( | ± | 1.42 | ) | 0.8471 |
| **LVPWd** (cm) | 0.815 | ( | ± | 0.145 | ) | 0.811 | ( | ± | 0.198 | ) | 1.14 | ( | ± | 0.85 | ) | 0.9301 |
| **LVPWs** (cm) | 1.144 | ( | ± | 0.232 | ) | 1.043 | ( | ± | 0.246 | ) | |  |  |  |  | 0.1506 |
| **FS** (%) | 32.32 | ( | ± | 7.35 | ) | 32.85 | ( | ± | 4.15 | ) | |  |  |  |  | 0.7454 |
| **FE** (%) | 61.19 | ( | ± | 0.1 | ) | 62.78 | ( | ± | 0.07 | ) | |  |  |  |  | 0.5421 |
| **DECEL TIME** (s) | 0.170 | ( | ± | 0.078 | ) | 0.177 | ( | ± | 0.052 | ) | |  |  |  |  | 0.7559 |
| **IVRT** (s) | 0.095 | ( | ± | 0.027 | ) | 0.096 | ( | ± | 0.021 | ) | |  |  |  |  | 0.8991 |
| **E** (m/s) | 0.864 | ( | ± | 0.161 | ) | 0.868 | ( | ± | 0.135 | ) | |  |  |  |  | 0.9246 |
| **A** (m/s) | 0.486 | ( | ± | 0.110 | ) | 0.504 | ( | ± | 0.117 | ) | |  |  |  |  | 0.6082 |
| **E/A RATIO** | 1.87 | ( | ± | 0.44 | ) | 1.78 | ( | ± | 0.40 | ) | |  |  |  |  | 0.4603 |
| *LVPWm* |  |  |  |  |  |  |  |  |  |  |  |  |  |  |  |  |
| **S** (m/s) | 0.098 | ( | ± | 0.03 | ) | 0.093 | ( | ± | 0.03 | ) | |  |  |  |  | 0.5206 |
| **E'** (m/s) | 0.159 | ( | ± | 0.033 | ) | 0.147 | ( | ± | 0.023 | ) | |  |  |  |  | 0.1675 |
| **A'** (m/s) | 0.044 | ( | ± | 0.012 | ) | 0.046 | ( | ± | 0.014 | ) | |  |  |  |  | 0.7075 |
| **E'/A' RATIO** | 3.842 | ( | ± | 1.328 | ) | 3.470 | ( | ± | 1.007 | ) | |  |  |  |  | 0.2997 |
| **E/E' RATIO** | 5.628 | ( | ± | 1.673 | ) | 5.797 | ( | ± | 0.841 | ) | |  |  |  |  | 0.6529 |
| *LVPWb* |  |  |  |  |  |  |  |  |  |  |  |  |  |  |  |  |
| **S** (m/s) | 0.105 | ( | ± | 0.028 | ) | 0.096 | ( | ± | 0.030 | ) | |  |  |  |  | 0.2361 |
| **E'** (m/s) | 0.180 | ( | ± | 0.022 | ) | 0.182 | ( | ± | 0.025 | ) | |  |  |  |  | 0.7616 |
| **A'** (m/s) | 0.054 | ( | ± | 0.016 | ) | 0.053 | ( | ± | 0.020 | ) | |  |  |  |  | 0.9173 |
| **E'/A' RATIO** | 3.559 | ( | ± | 0.902 | ) | 3.855 | ( | ± | 1.322 | ) | |  |  |  |  | 0.3521 |
| **E/E' RATIO** | 4.853 | ( | ± | 1.136 | ) | 4.685 | ( | ± | 0.666 | ) | |  |  |  |  | 0.5302 |
| *IVSm* |  |  |  |  |  |  |  |  |  |  |  |  |  |  |  |  |
| **S** (m/s) | 0.059 | ( | ± | 0.008 | ) | 0.058 | ( | ± | 0.012 | ) | |  |  |  |  | 0.5543 |
| **E'** (m/s) | 0.107 | ( | ± | 0.022 | ) | 0.105 | ( | ± | 0.026 | ) | |  |  |  |  | 0.7652 |
| **A'** (m/s) | 0.043 | ( | ± | 0.010 | ) | 0.039 | ( | ± | 0.014 | ) | |  |  |  |  | 0.2714 |
| **E'/A' RATIO** | 2.604 | ( | ± | 0.786 | ) | 2.870 | ( | ± | 0.977 | ) | |  |  |  |  | 0.2948 |
| **E/E' RATIO** | 8.484 | ( | ± | 2.590 | ) | 8.529 | ( | ± | 2.488 | ) | |  |  |  |  | 0.9544 |
| *IVSb* |  |  |  |  |  |  |  |  |  |  |  |  |  |  |  |  |
| **S** (m/s) | 0.074 | ( | ± | 0.013 | ) | 0.074 | ( | ± | 0.015 | ) | |  |  |  |  | 0.9256 |
| **E'** (m/s) | 0.126 | ( | ± | 0.026 | ) | 0.132 | ( | ± | 0.017 | ) | |  |  |  |  | 0.3859 |
| **A'** (m/s) | 0.050 | ( | ± | 0.014 | ) | 0.046 | ( | ± | 0.009 | ) | |  |  |  |  | 0.1869 |
| **E'/A' RATIO** | 2.723 | ( | ± | 1.008 | ) | 3.005 | ( | ± | 0.805 | ) | |  |  |  |  | 0.3074 |
| **E/E' RATIO** | 7.130 | ( | ± | 2.244 | ) | 6.544 | ( | ± | 0.968 | ) | |  |  |  |  | 0.2272 |
| *MAD* |  |  |  |  |  |  |  |  |  |  |  |  |  |  |  |  |
| **S** (m/s) | 0.077 | ( | ± | 0.023 | ) | 0.077 | ( | ± | 0.020 | ) | |  |  |  |  | 0.8799 |
| **E'** (m/s) | 0.140 | ( | ± | 0.031 | ) | 0.151 | ( | ± | 0.030 | ) | |  |  |  |  | 0.2254 |
| **A'** (m/s) | 0.051 | ( | ± | 0.020 | ) | 0.048 | ( | ± | 0.020 | ) | |  |  |  |  | 0.5797 |
| **E'/A' RATIO** | 3.183 | ( | ± | 1.497 | ) | 3.754 | ( | ± | 2.043 | ) | |  |  |  |  | 0.2647 |
| **E/E' RATIO** | 6.381 | ( | ± | 2.092 | ) | 5.621 | ( | ± | 1.071 | ) | |  |  |  |  | 0.11590 |

MAD: mitral annular displacement; LVPWm middle left ventricular posterior wall; LVPWb: basal left ventricular posterior wall; IVSm: middle interventricular septum; IVSb: basal interventricular septum.

**Table S8: t-test results and Z-scores between different time of TDI evaluation during follow-up.**

|  | **FUP ≤ 12 MONTHS** | | | | | **FUP > 12 MONTHS** | | | | | **FUP ≤ 12 m Z-score** | | | | |  |
| --- | --- | --- | --- | --- | --- | --- | --- | --- | --- | --- | --- | --- | --- | --- | --- | --- |
|  | mean values (± SD) | | | | | mean values (± SD) | | | | | mean values (± SD) | | | | | **P values** |
|  |  |  |  |  |  |  |  |  |  |  |  |  |  |  |  |  |
| **IVSd** (cm) | 0.868 | ( | ± | 0.099 | ) | 0.861 | ( | ± | 0.158 | ) | 2.13 | ( | ± | 1.03 | ) | 0.8945 |
| **IVSs** (cm) | 1.189 | ( | ± | 0.231 | ) | 1.140 | ( | ± | 0.256 | ) | |  |  |  |  | 0.5430 |
| **LVIDd** (cm) | 3.789 | ( | ± | 0.446 | ) | 4.068 | ( | ± | 0.528 | ) | -1.28 | ( | ± | 1.22 | ) | 0.0960 |
| **LVIDs** (cm) | 2.452 | ( | ± | 0.386 | ) | 2.779 | ( | ± | 0.423 | ) | -0.72 | ( | ± | 1.05 | ) |  |
| **LVPWd** (cm) | 0.822 | ( | ± | 0.120 | ) | 0.810 | ( | ± | 0.180 | ) | 1.20 | ( | ± | 0.94 | ) | 0.8253 |
| **LVPWs** (cm) | 1.211 | ( | ± | 0.302 | ) | 1.069 | ( | ± | 0.206 | ) | |  |  |  |  | 0.0658 |
| **FS** (%) | 35.32 | ( | ± | 6.37 | ) | 31.535 | ( | ± | 6.03 | ) | |  |  |  |  | 0.0613 |
| **FE** (%) | 0.67 | ( | ± | 0.09 | ) | 0.601 | ( | ± | 0.10 | ) | |  |  |  |  | 0.0486 |
| **DECEL TIME** (s) | 0.217 | ( | ± | 0.101 | ) | 0.161 | ( | ± | 0.052 | ) | |  |  |  |  | 0.1433 |
| **IVRT** (s) | 0.088 | ( | ± | 0.021 | ) | 0.098 | ( | ± | 0.026 | ) | |  |  |  |  | 0.2792 |
| **E** (m/s) | 0.891 | ( | ± | 0.135 | ) | 0.857 | ( | ± | 0.156 | ) | |  |  |  |  | 0.5216 |
| **A** (m/s) | 0.475 | ( | ± | 0.121 | ) | 0.498 | ( | ± | 0.110 | ) | |  |  |  |  | 0.5268 |
| **E/A RATIO** | 1.900 | ( | ± | 0.34 | ) | 1.816 | ( | ± | 0.45 | ) | |  |  |  |  | 0.4818 |
| *LVPWm* |  |  |  |  |  |  |  |  |  |  |  |  |  |  |  |  |
| **S** (m/s) | 0.104 | ( | ± | 0.03 | ) | 0.093 | ( | ± | 0.03 | ) | |  |  |  |  | 0.3190 |
| **E'** (m/s) | 0.156 | ( | ± | 0.03 | ) | 0.154 | ( | ± | 0.03 | ) | |  |  |  |  | 0.8848 |
| **A'** (m/s) | 0.043 | ( | ± | 0.013 | ) | 0.046 | ( | ± | 0.013 | ) | |  |  |  |  | 0.5816 |
| **E'/A' RATIO** | 3.872 | ( | ± | 1.379 | ) | 3.641 | ( | ± | 1.172 | ) | |  |  |  |  | 0.5615 |
| **E/E' RATIO** | 5.974 | ( | ± | 1.431 | ) | 5.601 | ( | ± | 1.418 | ) | |  |  |  |  | 0.4518 |
| *LVPWb* |  |  |  |  |  |  |  |  |  |  |  |  |  |  |  |  |
| **S** (m/s) | 0.088 | ( | ± | 0.030 | ) | 0.106 | ( | ± | 0.027 | ) | |  |  |  |  | 0.0792 |
| **E'** (m/s) | 0.180 | ( | ± | 0.02 | ) | 0.181 | ( | ± | 0.02 | ) | |  |  |  |  | 0.9341 |
| **A'** (m/s) | 0.050 | ( | ± | 0.012 | ) | 0.055 | ( | ± | 0.019 | ) | |  |  |  |  | 0.3846 |
| **E'/A' RATIO** | 3.787 | ( | ± | 1.002 | ) | 3.631 | ( | ± | 1.113 | ) | |  |  |  |  | 0.6583 |
| **E/E' RATIO** | 5.078 | ( | ± | 0.985 | ) | 4.701 | ( | ± | 0.980 | ) | |  |  |  |  | 0.2719 |
| *IVSm* |  |  |  |  |  |  |  |  |  |  |  |  |  |  |  |  |
| **S** (m/s) | 0.062 | ( | ± | 0.014 | ) | 0.057 | ( | ± | 0.008 | ) | |  |  |  |  | 0.3291 |
| **E'** (m/s) | 0.112 | ( | ± | 0.022 | ) | 0.104 | ( | ± | 0.024 | ) | |  |  |  |  | 0.2650 |
| **A'** (m/s) | 0.041 | ( | ± | 0.01 | ) | 0.042 | ( | ± | 0.01 | ) | |  |  |  |  | 0.7442 |
| **E'/A' RATIO** | 2.877 | ( | ± | 0.827 | ) | 2.644 | ( | ± | 0.879 | ) | |  |  |  |  | 0.4085 |
| **E/E' RATIO** | 8.294 | ( | ± | 2.601 | ) | 8.566 | ( | ± | 2.536 | ) | |  |  |  |  | 0.7596 |
| *IVSb* |  |  |  |  |  |  |  |  |  |  |  |  |  |  |  |  |
| **S** (m/s) | 0.078 | ( | ± | 0.017 | ) | 0.072 | ( | ± | 0.012 | ) | |  |  |  |  | 0.2531 |
| **E'** (m/s) | 0.135 | ( | ± | 0.018 | ) | 0.126 | ( | ± | 0.024 | ) | |  |  |  |  | 0.1907 |
| **A'** (m/s) | 0.048 | ( | ± | 0.019 | ) | 0.049 | ( | ± | 0.010 | ) | |  |  |  |  | 0.8080 |
| **E'/A' RATIO** | 3.166 | ( | ± | 1.08 | ) | 2.712 | ( | ± | 0.87 | ) | |  |  |  |  | 0.1353 |
| **E/E' RATIO** | 6.650 | ( | ± | 1.336 | ) | 6.996 | ( | ± | 2.035 | ) | |  |  |  |  | 0.6008 |
| *MAD* |  |  |  |  |  |  |  |  |  |  |  |  |  |  |  |  |
| **S** (m/s) | 0.079 | ( | ± | 0.020 | ) | 0.077 | ( | ± | 0.022 | ) | |  |  |  |  | 0.9096 |
| **E'** (m/s) | 0.143 | ( | ± | 0.037 | ) | 0.145 | ( | ± | 0.029 | ) | |  |  |  |  | 0.7964 |
| **A'** (m/s) | 0.047 | ( | ± | 0.016 | ) | 0.051 | ( | ± | 0.021 | ) | |  |  |  |  | 0.4707 |
| **E'/A' RATIO** | 3.271 | ( | ± | 1.141 | ) | 3.453 | ( | ± | 1.912 | ) | |  |  |  |  | 0.6878 |
| **E/E' RATIO** | 6.556 | ( | ± | 2.54 | ) | 5.944 | ( | ± | 1.51 | ) | |  |  |  |  | 0.33260 |

MAD: mitral annular displacement; LVPWm middle left ventricular posterior wall; LVPWb: basal left ventricular posterior wall; IVSm: middle interventricular septum; IVSb: basal interventricular septum.

**Table S9: t-test results and Z-scores between different time of TDI evaluation during follow-up.**

|  | **FUP ≤ 24 MONTHS** | | | | | **FUP > 24 MONTHS** | | | | | **FUP ≤ 24 m Z-score** | | | | |  |
| --- | --- | --- | --- | --- | --- | --- | --- | --- | --- | --- | --- | --- | --- | --- | --- | --- |
|  | mean values (± SD) | | | | | mean values (± SD) | | | | | mean values (± SD) | | | | | **P values** |
|  |  |  |  |  |  |  |  |  |  |  |  |  |  |  |  |  |
| **IVSd** (cm) | 0.883 | ( | ± | 0.153 | ) | 0.839 | ( | ± | 0.132 | ) | 2.28 | ( | ± | 1.53 | ) | 0.2893 |
| **IVSs** (cm) | 1.182 | ( | ± | 0.207 | ) | 1.119 | ( | ± | 0.290 | ) | |  |  |  |  | 0.3747 |
| **LVIDd** (cm) | 3.886 | ( | ± | 0.523 | ) | 4.123 | ( | ± | 0.494 | ) | -0.93 | ( | ± | 1.31 | ) | 0.1077 |
| **LVIDs** (cm) | 2.597 | ( | ± | 0.420 | ) | 2.807 | ( | ± | 0.433 | ) | -0.22 | ( | ± | 1.15 | ) |  |
| **LVPWd** (cm) | 0.798 | ( | ± | 0.136 | ) | 0.831 | ( | ± | 0.196 | ) | 1.02 | ( | ± | 0.89 | ) | 0.4910 |
| **LVPWs** (cm) | 1.118 | ( | ± | 0.259 | ) | 1.092 | ( | ± | 0.221 | ) | |  |  |  |  | 0.7091 |
| **FS** (%) | 33.36 | ( | ± | 5.343 | ) | 31.526 | ( | ± | 7.23 | ) | |  |  |  |  | 0.3076 |
| **FE** (%) | 0.63 | ( | ± | 0.075 | ) | 0.599 | ( | ± | 0.12 | ) | |  |  |  |  | 0.2456 |
| **DECEL TIME** (s) | 0.180 | ( | ± | 0.081 | ) | 0.166 | ( | ± | 0.054 | ) | |  |  |  |  | 0.5157 |
| **IVRT** (s) | 0.087 | ( | ± | 0.017 | ) | 0.104 | ( | ± | 0.028 | ) | |  |  |  |  | 0.0216 |
| **E** (m/s) | 0.884 | ( | ± | 0.134 | ) | 0.847 | ( | ± | 0.166 | ) | |  |  |  |  | 0.4149 |
| **A** (m/s) | 0.486 | ( | ± | 0.105 | ) | 0.499 | ( | ± | 0.120 | ) | |  |  |  |  | 0.7010 |
| **E/A RATIO** | 1.856 | ( | ± | 0.370 | ) | 1.813 | ( | ± | 0.47 | ) | |  |  |  |  | 0.6678 |
| *LVPWm* |  |  |  |  |  |  |  |  |  |  |  |  |  |  |  |  |
| **S** (m/s) | 0.102 | ( | ± | 0.026 | ) | 0.089 | ( | ± | 0.03 | ) | |  |  |  |  | 0.0954 |
| **E'** (m/s) | 0.150 | ( | ± | 0.030 | ) | 0.160 | ( | ± | 0.03 | ) | |  |  |  |  | 0.2409 |
| **A'** (m/s) | 0.045 | ( | ± | 0.014 | ) | 0.045 | ( | ± | 0.01 | ) | |  |  |  |  | 0.9087 |
| **E'/A' RATIO** | 3.585 | ( | ± | 1.205 | ) | 3.837 | ( | ± | 1.247 | ) | |  |  |  |  | 0.4726 |
| **E/E' RATIO** | 5.924 | ( | ± | 1.227 | ) | 5.457 | ( | ± | 1.572 | ) | |  |  |  |  | 0.2673 |
| *LVPWb* |  |  |  |  |  |  |  |  |  |  |  |  |  |  |  |  |
| **S** (m/s) | 0.100 | ( | ± | 0.031 | ) | 0.103 | ( | ± | 0.026 | ) | |  |  |  |  | 0.9336 |
| **E'** (m/s) | 0.180 | ( | ± | 0.025 | ) | 0.182 | ( | ± | 0.02 | ) | |  |  |  |  | 0.7805 |
| **A'** (m/s) | 0.053 | ( | ± | 0.016 | ) | 0.055 | ( | ± | 0.019 | ) | |  |  |  |  | 0.7616 |
| **E'/A' RATIO** | 3.667 | ( | ± | 1.063 | ) | 3.676 | ( | ± | 1.12 | ) | |  |  |  |  | 0.9775 |
| **E/E' RATIO** | 4.878 | ( | ± | 0.879 | ) | 4.704 | ( | ± | 1.092 | ) | |  |  |  |  | 0.5557 |
| *IVSm* |  |  |  |  |  |  |  |  |  |  |  |  |  |  |  |  |
| **S** (m/s) | 0.058 | ( | ± | 0.012 | ) | 0.059 | ( | ± | 0.006 | ) | |  |  |  |  | 0.3650 |
| **E'** (m/s) | 0.110 | ( | ± | 0.025 | ) | 0.102 | ( | ± | 0.021 | ) | |  |  |  |  | 0.2192 |
| **A'** (m/s) | 0.041 | ( | ± | 0.012 | ) | 0.042 | ( | ± | 0.01 | ) | |  |  |  |  | 0.6714 |
| **E'/A' RATIO** | 2.842 | ( | ± | 0.886 | ) | 2.544 | ( | ± | 0.826 | ) | |  |  |  |  | 0.2279 |
| **E/E' RATIO** | 8.381 | ( | ± | 2.626 | ) | 8.621 | ( | ± | 2.474 | ) | |  |  |  |  | 0.7511 |
| *IVSb* |  |  |  |  |  |  |  |  |  |  |  |  |  |  |  |  |
| **S** (m/s) | 0.075 | ( | ± | 0.017 | ) | 0.072 | ( | ± | 0.01 | ) | |  |  |  |  | 0.6231 |
| **E'** (m/s) | 0.133 | ( | ± | 0.018 | ) | 0.123 | ( | ± | 0.026 | ) | |  |  |  |  | 0.1134 |
| **A'** (m/s) | 0.048 | ( | ± | 0.014 | ) | 0.049 | ( | ± | 0.011 | ) | |  |  |  |  | 0.8284 |
| **E'/A' RATIO** | 2.962 | ( | ± | 0.890 | ) | 2.676 | ( | ± | 0.99 | ) | |  |  |  |  | 0.2872 |
| **E/E' RATIO** | 6.586 | ( | ± | 1.039 | ) | 7.240 | ( | ± | 2.440 | ) | |  |  |  |  | 0.2462 |
| *MAD* |  |  |  |  |  |  |  |  |  |  |  |  |  |  |  |  |
| **S** (m/s) | 0.076 | ( | ± | 0.020 | ) | 0.079 | ( | ± | 0.024 | ) | |  |  |  |  | 0.6807 |
| **E'** (m/s) | 0.144 | ( | ± | 0.034 | ) | 0.146 | ( | ± | 0.028 | ) | |  |  |  |  | 0.7929 |
| **A'** (m/s) | 0.050 | ( | ± | 0.021 | ) | 0.051 | ( | ± | 0.02 | ) | |  |  |  |  | 0.8622 |
| **E'/A' RATIO** | 3.475 | ( | ± | 1.917 | ) | 3.318 | ( | ± | 1.513 | ) | |  |  |  |  | 0.7556 |
| **E/E' RATIO** | 6.247 | ( | ± | 1.910 | ) | 5.933 | ( | ± | 1.71 | ) | |  |  |  |  | 0.5650 |

MAD: mitral annular displacement; LVPWm middle left ventricular posterior wall; LVPWb: basal left ventricular posterior wall; IVSm: middle interventricular septum; IVSb: basal interventricular septum.
