# Supplementary material for: Tissue Doppler Imaging for anthracycline cardiotoxicity monitoring in pediatric patients with cancer
Source: Cardiooncology. 2018 Sep 3;4:6. doi: 10.1186/s40959-018-0032-3 (PMC7048119; doi:10.1186/s40959-018-0032-3)
Supplement: Supplementary file 2 — Tables of linear regression analysis and multivariate analysis. Due to the large amount of data which would have been impractical to attach to the end of this manuscript, the authors provides this file with seventeen detailed tables (from “Table 10” to “Table 26”) supporting the results of the analysis. (DOCX 46 kb) [file 40959_2018_32_MOESM2_ESM.docx]

**Table S10: linear regression analysis results for middle left ventricular posterior wall (LVPWm) S wave.**

| INDEPENDENT variables | DEPENDENT variable | P values | β values |
| --- | --- | --- | --- |
|  |  |  |  |
| STOP TP - ECHO | S wave, LVPWm | 0.221 | -0.1763572 |
| DIAGNOSIS - ECHO | S wave, LVPWm | 0.804 | -0.0359065 |
| GENDER | S wave, LVPWm | 0.292 | 0.1519559 |
| BSA | S wave, LVPWm | 0.110 | 0.2290222 |
| MEDIASTINIC IRRADIATION | S wave, LVPWm | 0.642 | -0.0674446 |
| ANTHRACYCLINE EQUIVALENT DOSE | S wave, LVPWm | 0.163 | -0.2001777 |
| HSCT | S wave, LVPWm | 0.894 | 0.019869 |
| TUMOR TYPE | S wave, LVPWm | 0.521 | -0.093012 |

**Table S11: linear regression analysis results for middle left ventricular posterior wall (LVPWm) E’ wave.**

| INDEPENDENT variables | DEPENDENT variable | P values | β values |
| --- | --- | --- | --- |
|  |  |  |  |
| STOP TP - ECHO | E’ wave, LVPWm | 0.392 | -0.123788 |
| DIAGNOSIS - ECHO | E’ wave, LVPWm | 0.794 | -0.0378513 |
| SEX | E’ wave, LVPWm | 0.402 | -0.1211 |
| BSA | E’ wave, LVPWm | 0.776 | -0.0412716 |
| MEDIASTINIC IRRADIATION | E’ wave, LVPWm | 0.806 | 0.035674 |
| ANTHRACYCLINE EQUIVALENT DOSE | E’ wave, LVPWm | 0.714 | -0.0530819 |
| HSCT | E’ wave, LVPWm | 0.710 | -0.053844 |
| TUMOR TYPE | E’ wave, LVPWm | 0.168 | -0.1982523 |

**Table S12: linear regression analysis results for middle left ventricular posterior wall (LVPWm) E’ wave.**

| INDEPENDENT variables | DEPENDENT variable | P values | β values |
| --- | --- | --- | --- |
|  |  |  |  |
| STOP TP - ECHO | A' wave, LVPWm | 0.295 | -0.1510911 |
| DIAGNOSIS - ECHO | A' wave, LVPWm | 0.983 | -0.0030208 |
| SEX | A' wave, LVPWm | 0.339 | 0.1380117 |
| BSA | A' wave, LVPWm | 0.238 | -0.1700163 |
| MEDIASTINIC IRRADIATION | A' wave, LVPWm | 0.145 | 0.2089828 |
| ANTHRACYCLINE EQUIVALENT DOSE | A' wave, LVPWm | 0.741 | -0.0478864 |
| HSCT | A' wave, LVPWm | 0.560 | 0.0843462 |
| TUMOR TYPE | A' wave, LVPWm | 0.707 | 0.054407 |

**Table S13: linear regression analysis results for basal left ventricular posterior wall (LVPWb) S wave.**

| INDEPENDENT variables | DEPENDENT variable | P values | β values |
| --- | --- | --- | --- |
|  |  |  |  |
| STOP TP - ECHO | S wave, LVPWb | 0.900 | -0.0181901 |
| DIAGNOSIS - ECHO | S wave, LVPWb | 0.090 | 0.2425451 |
| SEX | S wave, LVPWb | 0.736 | 0.0489389 |
| BSA | S wave, LVPWb | 0.121 | 0.2223253 |
| MEDIASTINIC IRRADIATION | S wave, LVPWb | 0.569 | 0.0824411 |
| ANTHRACYCLINE EQUIVALENT DOSE | S wave, LVPWb | 0.615 | 0.0728691 |
| HSCT | S wave, LVPWb | 0.743 | 0.0475931 |
| TUMOR TYPE | S wave, LVPWb | 0.236 | -0.1706332 |

**Table S14: linear regression analysis results for basal left ventricular posterior wall (LVPWb) E’ wave, and multivariate analysis for statistically significant values.**

| INDEPENDENT variables | DEPENDENT variable | P values | β values | **MULTIVARIATE ANALYSIS** | |
| --- | --- | --- | --- | --- | --- |
|  |  |  |  | P values | β values |
| STOP TP - ECHO | E' wave, LVPWb | 0.893 | -0.019493 |  |  |
| DIAGNOSIS - ECHO | E' wave, LVPWb | 0.691 | 0.0576199 |  |  |
| SEX | E' wave, LVPWb | 0.638 | -0.0682501 |  |  |
| BSA | E' wave, LVPWb | 0.383 | 0.1259723 |  |  |
| MEDIASTINIC IRRADIATION | E' wave, LVPWb | 0.781 | 0.040228 |  |  |
| ANTHRACYCLINE EQUIVALENT DOSE | E' wave, LVPWb | 0.040 | -0.2916405 | 0.065 | -0.2478922 |
| HSCT | E' wave, LVPWb | 0.006 | -0.3810903 | 0.010 | -0.3501152 |
| TUMOR TYPE | E' wave, LVPWb | 0.762 | 0.0439948 |  |  |

**Table S15: linear regression analysis results for basal left ventricular posterior wall (LVPWb) A’ wave.**

| INDEPENDENT variables | DEPENDENT variable | P values | β values |
| --- | --- | --- | --- |
|  |  |  |  |
| STOP TP - ECHO | A' wave, LVPWb | 0.465 | -0.1056712 |
| DIAGNOSIS - ECHO | A' wave, LVPWb | 0.695 | 0.0565862 |
| SEX | A' wave, LVPWb | 0.648 | -0.0662358 |
| BSA | A' wave, LVPWb | 0.271 | -0.1585459 |
| MEDIASTINIC IRRADIATION | A' wave, LVPWb | 0.189 | 0.1890727 |
| ANTHRACYCLINE EQUIVALENT DOSE | A' wave, LVPWb | 0.402 | -0.1211695 |
| HSCT | A' wave, LVPWb | 0.354 | 0.133946 |
| TUMOR TYPE | A' wave, LVPWb | 0.917 | -0.0150604 |

**Table S16: linear regression analysis results for middle interventricular septum(IVSm) E’ wave.**

| INDEPENDENT variables | DEPENDENT variable | P values | β values |
| --- | --- | --- | --- |
|  |  |  |  |
| STOP TP - ECHO | E' wave, IVSm | 0.082 | -0.2486473 |
| DIAGNOSIS - ECHO | E' wave, IVSm | 0.133 | -0.2152285 |
| SEX | E' wave, IVSm | 0.865 | 0.0246415 |
| BSA | E' wave, IVSm | 0.962 | -0.0069644 |
| MEDIASTINIC IRRADIATION | E' wave, IVSm | 0.652 | 0.0654437 |
| ANTHRACYCLINE EQUIVALENT DOSE | E' wave, IVSm | 0.230 | -0.1728725 |
| HSCT | E' wave, IVSm | 0.011 | -0.3572392 |
| TUMOR TYPE | E' wave, IVSm | 0.765 | -0.0433049 |

**Table S17: linear regression analysis results for middle interventricular septum(IVSm) E’ wave.**

| INDEPENDENT variables | DEPENDENT variable | P values | β values |
| --- | --- | --- | --- |
|  |  |  |  |
| STOP TP - ECHO | A' wave, IVSm | 0.620 | -0.0719318 |
| DIAGNOSIS - ECHO | A' wave, IVSm | 0.597 | -0.0766483 |
| SEX | A' wave, IVSm | 0.197 | -0.1855363 |
| BSA | A' wave, IVSm | 0.292 | 0.1519638 |
| MEDIASTINIC IRRADIATION | A' wave, IVSm | 0.285 | -0.1540475 |
| ANTHRACYCLINE EQUIVALENT DOSE | A' wave, IVSm | 0.264 | -0.1611151 |
| HSCT | A' wave, IVSm | 0.273 | 0.1580388 |
| TUMOR TYPE | A' wave, IVSm | 0.271 | -0.1585812 |

**Table S18: linear regression analysis results for basal interventricular septum (IVSb) E’ wave.**

| INDEPENDENT variables | DEPENDENT variable | P values | β values |
| --- | --- | --- | --- |
|  |  |  |  |
| STOP TP - ECHO | S wave, IVSb | 0.231 | -0.172514 |
| DIAGNOSIS - ECHO | S wave, IVSb | 0.231 | -0.1724383 |
| SEX | S wave, IVSb | 0.498 | -0.0979866 |
| BSA | S wave, IVSb | 0.917 | 0.0151338 |
| MEDIASTINIC IRRADIATION | S wave, IVSb | 0.247 | -0.1668157 |
| ANTHRACYCLINE EQUIVALENT DOSE | S wave, IVSb | 0.025 | -0.3162715 |
| HSCT | S wave, IVSb | 0.161 | 0.2014474 |
| TUMOR TYPE | S wave, IVSb | 0.926 | -0.0135512 |

**Table S19: linear regression analysis results for basal interventricular septum (IVSb) E’ wave.**

| INDEPENDENT variables | DEPENDENT variable | P values | β values | **MULTIVARIATE ANALYSIS** | |
| --- | --- | --- | --- | --- | --- |
|  |  |  |  | P values | β values |
| STOP TP - ECHO | E' wave, IVSb | 0.024 | -0.3197128 | 0.020 | -0.3156826 |
| DIAGNOSIS - ECHO | E' wave, IVSb | 0.068 | -0.2600953 |  |  |
| SEX | E' wave, IVSb | 0.076 | -0.2531033 |  |  |
| BSA | E' wave, IVSb | 0.402 | 0.1211272 |  |  |
| MEDIASTINIC IRRADIATION | E' wave, IVSb | 0.871 | 0.0235554 |  |  |
| ANTHRACYCLINE EQUIVALENT DOSE | E' wave, IVSb | 0.094 | -0.2396219 |  |  |
| HSCT | E' wave, IVSb | 0.028 | -0.3104282 | 0.023 | -0.3062743 |
| TUMOR TYPE | E' wave, IVSb | 0.432 | 0.1135493 |  |  |

**Table S20: linear regression analysis results for basal interventricular septum (IVSb) A’ wave.**

| INDEPENDENT variables | DEPENDENT variable | P values | β values |
| --- | --- | --- | --- |
|  |  |  |  |
| STOP TP - ECHO | A' wave, IVSb | 0.509 | -0.0955619 |
| DIAGNOSIS - ECHO | A' wave, IVSb | 0.842 | -0.0288974 |
| SEX | A' wave, IVSb | 0.275 | -0.1573599 |
| BSA | A' wave, IVSb | 0.340 | -0.1376649 |
| MEDIASTINIC IRRADIATION | A' wave, IVSb | 0.785 | -0.0395726 |
| ANTHRACYCLINE EQUIVALENT DOSE | A' wave, IVSb | 0.641 | -0.0675682 |
| HSCT | A' wave, IVSb | 0.015 | 0.3417565 |
| TUMOR TYPE | A' wave, IVSb | 0.231 | -0.172441 |

**Table S21: linear regression analysis results for mitral annular displacement (MAD) E’ wave.**

| INDEPENDENT variables | DEPENDENT variable | P values | β values |
| --- | --- | --- | --- |
|  |  |  |  |
| STOP TP - ECHO | E' wave, MAD | 0.949 | 0.0094522 |
| DIAGNOSIS - ECHO | E' wave, MAD | 0.425 | 0.1164991 |
| SEX | E' wave, MAD | 0.512 | -0.0958817 |
| BSA | E' wave, MAD | 0.742 | 0.0482652 |
| MEDIASTINIC IRRADIATION | E' wave, MAD | 0.170 | 0.1992419 |
| ANTHRACYCLINE EQUIVALENT DOSE | E' wave, MAD | 0.250 | -0.1674012 |
| HSCT | E' wave, MAD | 0.215 | -0.180339 |
| TUMOR TYPE | E' wave, MAD | 0.225 | 0.1763645 |

**Table S22: linear regression analysis results for mitral annular displacement (MAD) A’ wave.**

| INDEPENDENT variables | DEPENDENT variable | P values | β values |
| --- | --- | --- | --- |
|  |  |  |  |
| STOP TP - ECHO | A' wave, MAD | 0.845 | -0.0286202 |
| DIAGNOSIS - ECHO | A' wave, MAD | 0.737 | 0.0492728 |
| SEX | A' wave, MAD | 0.534 | -0.0910068 |
| BSA | A' wave, MAD | 0.967 | 0.0060394 |
| MEDIASTINIC IRRADIATION | A' wave, MAD | 0.730 | 0.0505726 |
| ANTHRACYCLINE EQUIVALENT DOSE | A' wave, MAD | 0.688 | -0.0588007 |
| HSCT | A' wave, MAD | 0.853 | -0.0272378 |
| TUMOR TYPE | A' wave, MAD | 0.580 | -0.081086 |

**Table S23: linear regression analysis results for middle left ventricular posterior wall (LVPWm) E’/A’ ratio.**

| INDEPENDENT variables | DEPENDENT variable | P values | β values |
| --- | --- | --- | --- |
|  |  |  |  |
| STOP TP - ECHO | E'/A' ratio, LVPWm | 0.691 | 0.0576041 |
| DIAGNOSIS - ECHO | E'/A' ratio, LVPWm | 0.955 | -0.0082359 |
| SEX | E'/A' ratio, LVPWm | 0.505 | -0.0965088 |
| BSA | E'/A' ratio, LVPWm | 0.713 | 0.0533417 |
| MEDIASTINIC IRRADIATION | E'/A' ratio, LVPWm | 0.837 | -0.029899 |
| ANTHRACYCLINE EQUIVALENT DOSE | E'/A' ratio, LVPWm | 0.802 | 0.0363929 |
| HSCT | E'/A' ratio, LVPWm | 0.666 | 0.0625404 |
| TUMOR TYPE | E'/A' ratio, LVPWm | 0.300 | -0.1496263 |

**Table S24: linear regression analysis results for middle left ventricular posterior wall (LVpWm) E/E’ ratio.**

| INDEPENDENT variables | DEPENDENT variable | P values | β values |
| --- | --- | --- | --- |
|  |  |  |  |
| STOP TP - ECHO | E/E' ratio, LVPWm | 0.751 | 0.0481695 |
| DIAGNOSIS - ECHO | E/E' ratio, LVPWm | 0.854 | -0.027826 |
| SEX | E/E' ratio, LVPWm | 0.250 | 0.173015 |
| BSA | E/E' ratio, LVPWm | 0.797 | -0.0389715 |
| MEDIASTINIC IRRADIATION | E/E' ratio, LVPWm | 0.255 | 0.1714149 |
| ANTHRACYCLINE EQUIVALENT DOSE | E/E' ratio, LVPWm | 0.229 | 0.1808541 |
| HSCT | E/E' ratio, LVPWm | 0.703 | 0.0578479 |
| TUMOR TYPE | E/E' ratio, LVPWm | 0.701 | 0.0580862 |

**Table S25: linear regression analysis results for fractional shortening (FS).**

| INDEPENDENT variables | DEPENDENT variable | P values | β values |
| --- | --- | --- | --- |
|  |  |  |  |
| STOP TP - ECHO | FS | 0.323 | -0.1425359 |
| DIAGNOSIS - ECHO | FS | 0.254 | -0.1642624 |
| SEX | FS | 0.586 | -0.0789118 |
| BSA | FS | 0.323 | -0.1427086 |
| MEDIASTINIC IRRADIATION | FS | 0.802 | -0.0364505 |
| ANTHRACYCLINE EQUIVALENT DOSE | FS | 0.713 | -0.0534185 |
| HSCT | FS | 0.539 | -0.0888825 |
| TUMOR TYPE | FS | 0.775 | 0.0414824 |

**Table S26: linear regression analysis results for ejection fraction (EF).**

| INDEPENDENT variables | DEPENDENT variable | P values | β values |
| --- | --- | --- | --- |
|  |  |  |  |
| STOP TP - ECHO | EF | 0.492 | -0.0993669 |
| DIAGNOSIS - ECHO | EF | 0.503 | -0.0969112 |
| SEX | EF | 0.848 | -0.0278153 |
| BSA | EF | 0.292 | -0.1520705 |
| MEDIASTINIC IRRADIATION | EF | 0.759 | 0.0445714 |
| ANTHRACYCLINE EQUIVALENT DOSE | EF | 0.923 | -0.0140625 |
| HSCT | EF | 0.931 | -0.0126293 |
| TUMOR TYPE | EF | 0.592 | 0.0776345 |
